# Pathogenic mycobacteria achieve cellular persistence by inhibiting the Niemann-Pick Type C disease cellular pathway

**DOI:** 10.12688/wellcomeopenres.10036.2

**Published:** 2017-06-21

**Authors:** Paul Fineran, Emyr Lloyd-Evans, Nathan A. Lack, Nick Platt, Lianne C. Davis, Anthony J. Morgan, Doris Höglinger, Raju Venkata V. Tatituri, Simon Clark, Ian M. Williams, Patricia Tynan, Nada Al Eisa, Evgeniya Nazarova, Ann Williams, Antony Galione, Daniel S. Ory, Gurdyal S. Besra, David G. Russell, Michael B. Brenner, Edith Sim, Frances M. Platt

**Affiliations:** 1 Department of Pharmacology, University of Oxford, Oxford, UK; 2 School of Biosciences, Cardiff University, Cardiff, UK; 3 School of Medicine, Koç University, Istanbul, Turkey; 4 Department of Medicine, Brigham and Women's Hospital, Harvard Medical School, Boston, USA; 5 Public Health England, Salisbury, UK; 6 Department of Microbiology and Immunology, College of Veterinary Medicine, Cornell University, Ithaca, USA; 7 Diabetic Cardiovascular Disease Center, Washington University School of Medicine, St. Louis, USA; 8 School of Biosciences, University of Birmingham, Birmingham, UK; 9 Faculty of Science Engineering and Computing, Kingston University, Kingston upon Thames, UK

**Keywords:** Tuberculosis, Niemann-Pick Disease Type C, Lysosomal Storage Diseases, Lysosomal Calcium

## Abstract

***Background**. *Tuberculosis remains a major global health concern. The ability to prevent phagosome-lysosome fusion is a key mechanism by which intracellular mycobacteria, including *Mycobacterium tuberculosis*, achieve long-term persistence within host cells. The mechanisms underpinning this key intracellular pro-survival strategy remain incompletely understood. Host macrophages infected with intracellular mycobacteria share phenotypic similarities with cells taken from patients suffering from Niemann-Pick Disease Type C (NPC), a rare lysosomal storage disease in which endocytic trafficking defects and lipid accumulation within the lysosome lead to cell dysfunction and cell death. We investigated whether these shared phenotypes reflected an underlying mechanistic connection between mycobacterial intracellular persistence and the host cell pathway dysfunctional in NPC.

***Methods**. *The induction of NPC phenotypes in macrophages from wild-type mice or obtained from healthy human donors was assessed via infection with mycobacteria and subsequent measurement of lipid levels and intracellular calcium homeostasis. The effect of NPC therapeutics on intracellular mycobacterial load was also assessed.

***Results**. *Macrophages infected with intracellular mycobacteria phenocopied NPC cells, exhibiting accumulation of multiple lipid types, reduced lysosomal Ca ^2+ ^levels, and defects in intracellular trafficking. These NPC phenotypes could also be induced using only lipids/glycomycolates from the mycobacterial cell wall. These data suggest that intracellular mycobacteria inhibit the NPC pathway, likely via inhibition of the NPC1 protein, and subsequently induce altered acidic store Ca ^2+^ homeostasis. Reduced lysosomal calcium levels may provide a mechanistic explanation for the reduced levels of phagosome-lysosome fusion in mycobacterial infection. Treatments capable of correcting defects in NPC mutant cells via modulation of host cell calcium were of benefit in promoting clearance of mycobacteria from infected host cells.

***Conclusion**. *These findings provide a novel mechanistic explanation for mycobacterial intracellular persistence, and suggest that targeting interactions between the mycobacteria and host cell pathways may provide a novel avenue for development of anti-TB therapies.

## Introduction

Approximately one-third of the world’s population is infected with *Mycobacterium tuberculosis* ( *Mtb*), the causative agent of tuberculosis (TB). TB causes around 1.5 million deaths per year ^1^, a significant number of which are in immune-compromised individuals ^2^. The only approved vaccine, Bacillus Calmette-Guerin (BCG) has limited efficacy ^3^ and the emergence of antibiotic-resistant TB strains has led to a reduction in available therapeutic options. Consequently, the development of new TB therapies is of paramount importance.

Environmental mycobacteria, including *Mycobacterium smegmatis (Msm),* bind host cell-surface receptors and are ingested into phagosomes that subsequently mature and fuse with lysosomes, leading to the bacteria’s destruction. In contrast, intracellular mycobacteria, such as *Mtb* (and the *Mtb*-related attenuated vaccine strain *M.bovis* BCG), can inhibit phagosome-lysosome fusion and hence have the ability to invade, persist and replicate within cells of the innate immune system, particularly alveolar macrophages ^4^. *Mtb*-infected cells develop a cholesterol-laden foamy cell phenotype ^5^ and metabolise host cholesterol as a carbon source ^6– 8^. Multiple mechanisms have been proposed to explain how pathogenic mycobacterial species can block phagosome-lysosome fusion, including phagosome maturation arrest ^9, 10^, defective acidification ^11^ and inhibition of phosphatidylinositol-dependent trafficking pathways ^12, 13^. Calcium ions (Ca ^2+^) have also been implicated: Phagosome-lysosome fusion has been suggested to be stimulated by an elevation of cytosolic Ca ^2+^ ^14^, and a pharmacological elevation of host cell Ca ^2+^ was observed to lead to an increase in markers of phagosomal maturation and a decrease in the survival of intracellular mycobacteria ^15^. In *Mtb-*infected macrophages this Ca ^2+^ elevation is reduced, thereby blocking phagosome-lysosome fusion and facilitating mycobacterial survival within host cells ^15^. However, another study has indicated that phagosome-lysosome fusion may be a Ca ^2+^ independent process ^16^. Defects in phagosome-lysosome fusion, and failure to clear intracellular mycobacteria, mean that the infection can persist within the host for decades. The formation of a granuloma serves to isolate the infected macrophages and render the host asymptomatic and non-contagious (latent tuberculosis) ^17^. Individuals with latent TB still harbour the mycobacteria, and may progress to the active form of the disease in the future ^5^.

Cholesterol storage and failures in the fusion of late endosomes/lysosomes (LE/Lys) also occur in the lysosomal storage disease, Niemann-Pick type C (NPC) ^18^. NPC is caused by mutations in the *NPC1* (95% of clinical cases) or *NPC2* ^18^ genes, with defects in either gene resulting in identical clinical phenotypes. *NPC1* encodes NPC1, a membrane protein in the limiting LE/Lys membrane ^19^. In contrast, NPC2 is a soluble cholesterol-binding protein of the lysosomal lumen ^20^. It has been proposed that NPC1 and NPC2 exchange cholesterol, although whether the NPC pathway serves primarily to efflux cholesterol or is instead a cholesterol regulated/sensing pathway that effluxes/interacts with other substrates remains unresolved ^21^. Upon the pharmacological inactivation of NPC1 the first measurable event is an increase in sphingosine levels in the LE/Lys, rapidly followed by decreased lysosomal Ca ^2+^ levels and subsequent attenuated Ca ^2+^ release from the LE/Lys. This leads to downstream endocytic trafficking defects, failure in LE/Lys fusion ^22, 23^ and the subsequent storage of cholesterol and glycosphingolipids (GSLs) in a distended endo-lysosomal compartment. In addition to storage of multiple lipids, NPC cells also accumulate autophagic vacuoles, due to a failure in their clearance ^24, 25^. Many of these NPC cellular phenotypes ^21^ are also observed in *Mtb*-infected macrophages, including endocytic transport abnormalities, defective autophagy, accumulation of free cholesterol, elevated levels of GSLs and the presence of lamellar storage bodies ^4^. These shared phenotypes prompted us to investigate whether there is a mechanistic link between infection with intracellular mycobacteria and the host cell NPC pathway. We hypothesised that inhibition of the functional NPC pathway upon the infection of wild-type host cells, and the subsequent formation of an NPC-like cell with the associated defects in lysosomal Ca ^2+^ homeostasis and lysosomal fusion, could account for the defect in phagosome-lysosome fusion and the reduced mycobacterial clearance.

Here, we have found that infection with intracellular mycobacteria, such as BCG and TB, induced the full range of NPC phenotypes in wild-type cells, and lipids shed by these mycobacteria were able to phenocopy NPC disease cellular phenotypes in the absence of the mycobacteria itself. Furthermore, therapies developed for the treatment of NPC disease promoted mycobacterial clearance, suggesting novel host-targeted therapeutic approaches to treat mycobacterial infection, including TB.

## Methods

### Ethics statement

All experiments involving animals were conducted under the authority of project licence number PPL 30/2923, approved by the University of Oxford Animal Welfare and Ethical Review Body and granted by the United Kingdom Home Office. Animals were housed in the Biomedical Research Services facilities, University of Oxford. All licensed procedures were performed in accordance with the United Kingdom Animals (Scientific Procedures) Act 1986.

Human peripheral blood mononuclear cells used in this study were from healthy anonymous donors, and were isolated from buffy coats processed by and purchased from The National Blood and Transplantation Services, Bristol, UK.

### Cells

RAW 264.7 macrophages were obtained from the European Cell Culture Collection (Porton Down, UK). Bone marrow macrophages were isolated from 8-week-old mice and cultured at 37°C with 5% CO _2_ in RPMI with 10% foetal calf serum (FCS), 1% penicillin/streptomycin and 1% L-glutamine (Lonza, Basel, Switzerland). *Mtb* (H37Rv) and *M. bovis* BCG (Pasteur strain) were kindly provided by Simon Clark (Public Health England). Fluorescent *Msm* (mc ^2^155 strain expressing mCherry) was kindly provided by David Russell (Cornell University). Mycobacteria were grown on 7H11 agar plates (with Oleic Albumin Dextrose Catalase) before transfer to 7H9 liquid medium (with Albumin Dextrose Catalase). Mycobacterial cultures were maintained at 37 ^o^C, with shaking speed of 220rpm for liquid cultures. NPC1-overexpressing CHO cells ^26^ were kindly provided by Daniel Ory (Washington University School of Medicine) and were grown at 37°C with 5% CO _2_ in DMEM-F12, 10% FCS, 1% penicillin/streptomycin and 1% glutamine. U18666A (Sigma) was used at 1μg/ml for 48h. HeLa cells were obtained from ATCC and were kept in DMEM with low glucose (1g/L), 10% FCS and 1% primocin (InvivoGen). HEK293 cells were obtained from ATCC and were kept in DMEM with high glucose (4.5g/L) supplemented with 10% FCS and 1% penicillin/streptomycin.

### Human monocyte-derived macrophages

Peripheral blood CD14 ^+^ monocytes were isolated using microbeads (Miltenyi Biotec), differentiated in the presence of M-CSF (10ng/ml) in X-vivo media (Lonza) and used after 7 days.

### FLUOS labelling of mycobacteria

A small volume (5ml) of a mid-exponential (OD _600_ between 0.8 and 1.2) mycobacteria culture was centrifuged (3000g/10min), resuspended in 500μl of HEPES buffer (pH 9.1) and incubated for 5min with 25μl of 20mg/ml FLUOS (5(6)-carboxyfluorescein-N-hydroxysuccinimide ester) (Sigma) in DMSO. The bacteria were washed twice with warm 7H9 (37°C) and resuspended in 500μl of RPMI-FCS. The OD _600_ of the solution was measured via spectrophotometry (Jenway 6305 spectrophotometer) and the concentration of the bacteria was determined.

### Generation of mCherry-expressing BCG

BCG was electroporated with pV116 plasmid DNA (250–500ng) (kindly provided by David Russell, Cornell University) containing the gene for mCherry production and selective markers for kanamycin resistance, using standard parameters (Equibio Easyject Plus Eletroporator at 2.5kV, 25μF, 1000Ω). Transformed colonies were selected on 7H11 OADC agar plates supplemented with kanamycin. Individual colonies were picked and grown in liquid culture as detailed above.

### Host cell infection

The multiplicity of infection (MOI) used was 12.5. Host cells were plated out 18h prior to infection. Mid-log phase mycobacteria were centrifuged (3000g/10min) and resuspended in medium prior to dilution.

### Indirect calcium quantification

Cells were infected with mycobacteria or treated with lipids 24hr prior to Ca ^2+^ measurements. Cells were loaded with 2μM fura-2 AM (Teflabs), washed once with Ca ^2+^-free buffer [121 NaCl, 5.4 KCl, 0.8 MgCl _2_, 6 NaHCO _3_, 25 HEPES, 10 glucose (mM)] supplemented with 1mM ethylene glycol tetraacetic acid (EGTA) and twice with Ca ^2+^-free buffer containing 100μM EGTA; subsequent experiments were conducted in this same buffer. Cells were mounted on an Olympus IX71 microscope equipped with a 40x UApo/340 objective (1.35 NA) and a 12-bit Photometrics Coolsnap HQ2 CCD camera. Cells were excited alternately by 350- and 380-nm light using a Cairn monochromator; emission data were collected at 480–540 nm using a bandpass filter. Experiments were conducted at room temperature with an image collected every 2–3 seconds.

Lysosomal Ca ^2+^ release was assessed upon addition of 200μM glycyl-L-phenylalanine-β-napthylamide (GPN; Santa Cruz Biotechnology). At the end of each run, autofluorescence was determined by addition of 1μM ionomycin (Calbiochem) with 4mM MnCl _2_, which quenches fura-2. Images were analysed using custom-written Magipix software v3.02 (R. Jacob, King’s College London, UK) on a single-cell basis, the autofluorescence signal was subtracted and the data expressed as the mean ± SEM maximum fluorescence changes (Δ350/380).

### Direct calcium quantification

Calcium concentrations were quantified as described ^22^ with low-affinity Rhod-dextran ( *K*d=551 ± 107μM) (Invitrogen) in conjunction with the calcium-insensitive Alexa-Fluor 488 dextran (Invitrogen) at concentrations of 0.25mg/ml and 0.1mg/ml, respectively. Dextrans were loaded for 12hr, followed by a 12hr chase.

### Determination of lysosomal pH

RAW 264.7 cells were loaded with fluorescein (pH-insensitive) and Texas Red (pH-insensitive) dextrans (10,000MW; Thermo Fisher Scientific) at 0.2 mg/ml in complete RPMI in 96-well plates at 37 ^o^C for 16h. Cells were washed three times with dextran-free media and incubated for a further 7h to chase the dextrans to the lysosomes. Fluorescence measurements of labelled lysosomes were collected using a Novostar plate reader (BMG Labtech) using excitation/emission 485/520nm (fluorescein) and 570/620nm (Texas Red). For the calibration curves, lysosomal pH was set at the indicated values by equilibrating dextran-loaded cells in a high K ^+^ extracellular buffer [5 NaCl, 145 KCl, 1 MgCl _2_, 1 CaCl _2_, 10 glucose (mM)] and adjusted to a series of defined pH values in buffers (10mM acetate for pH 4–5; 10mM MES for pH 5.5 – 6.5; and 10mM HEPES for pH 7) containing 10μM nigericin and 10μM valinomycin (Sigma). Autofluorescence was subtracted and the fluorescein fluorescence (G) was divided by the Texas-Red fluorescence (R) and an *in situ* pH standard curve was constructed for both treatments [with cells maintained in normal medium, the resting G/R ratio of untreated (Ctrl) or BCG-treated cells was calibrated in terms of absolute pH].

### Indirect assessment of lysosomal cathepsin C activity

The lysosomes of RAW 264.7 macrophages, which had been infected with BCG mCherry for 24h, and control cells were labelled with 100nM LysoTracker Green DND-26 (Thermo Fisher Scientific) for 5min at room temperature in a buffer containing (mM): 121 NaCl, 5.4 KCl, 0.8 MgCl _2_, 1.8 CaCl _2_, 6 NaHCO _3_, 25 HEPES, 10 glucose. The cells were washed once in the same buffer, but without Ca ^2+^ (Ca ^2+^-free buffer), and supplemented with 1mM EGTA. The cells were then washed twice with Ca ^2+^-free buffer containing 100µM EGTA and subsequent experiments conducted in this buffer. The cells were mounted on the stage of a Zeiss LSM510 Meta confocal laser-scanning microscope equipped with a 40x objective; excitation/emission (nm): green (488/505–530), red (543/>560). Experiments were conducted at room temperature with an image collected every 1s. The activity of cathepsin C was assessed by the release of LysoTracker (i.e. a decrease in fluorescence) from lysosomes upon the addition of 200µM GPN. Images were analysed using custom-written Magipix software (R. Jacob, King’s College London, UK) on a single-cell basis. Data are presented as the mean ± SEM of the initial rate (units of LysoTracker fluorescence per second normalised to the basal fluorescence) and by the rate constant calculated from an exponential curve fit.

### Sphingosine HPLC measurement

Lipids were extracted as previously described ^27^ with the following modifications. Post-addition of 2ml 1:1 chloroform:methanol (C:M) samples were spiked with 1μl (1mM) C _20_ sphingosine standard (Avanti Polar Lipids). Solvent A was replaced with 1:1 MeOH:H _2_O and RP18 SPE 1ml columns (Supelco) were used for solid-phase extraction. Post-sample addition, columns were washed with 2×1ml 1:1 MeOH:H _2_O and 4×1ml 3:1 MeOH:H _2_O w/0.1% acetic acid, and the sample was eluted in 4×1ml 9:1 MeOH-10mM KH _2_PO _4_. The elutant was dried under N _2_ and resuspended in 1ml HPLC-grade EtOH, prior to drying down and resuspension in 50μl warm EtOH. Extracted sphingoid bases were labelled with 50μl orthophthaldehyde reagent (12.5mg orthophthaldehyde (Sigma-Aldrich), 12.5μl 2-mercaptoethanol (Sigma-Aldrich), 0.25ml EtOH, 24.75ml 3% boric acid (pH 10.5) and incubated for 5min at room temperature. Reverse Phase High Performance Liquid Chromatography (RP-HPLC) was carried out using a system consisting of a VWR Hitachi Organizer module, L-2200 Autosampler, L-2130 Pump, L-2485 FL Detector and BetaBasic-18 column (3μm; 100×4.6mm). Chromatography was carried out using a mobile phase of 85% acetonitrile/15% H2O at a flow rate of 1.0ml/min. The orthophthaldehyde-labelled derivatives were monitored at an excitation wavelength of 340nm and an emission wavelength of 450nm. Quantification of trace peak area was carried out using EZChrom Elite software v3.2.1 ( http://www.jascoinc.com/ezchrom).

### GSL HPLC measurement

GSLs were extracted from cellular homogenates (~200μg protein) in 4 volumes of C:M (1:2 v/v) overnight at room temperature. The mixture was centrifuged (1200g/10min) before the addition of 0.5ml chloroform and 0.5ml PBS to the supernatant, and a repetition of centrifugation (1200g/10min). The resulting lower phase was dried under N _2_, re-suspended in 50μl C:M 1:3 and recombined with the upper phase. GSLs were recovered using 25mg C18 Isolute columns (Biotage) pre-equilibrated with 4×1ml MeOH and 2×1ml H _2_O. The sample was eluted via 1ml C:M 98:2, 2×1ml C:M 1:3, 1ml MeOH. Column elutant was dried under N _2_ and re-suspended in 100μl C:M 2:1, before being dried down and re-suspended in ceramide glycanase (CGase) buffer. CGase (50mU) was added, and samples were incubated at 37 ^o^C for 16h. Released oligosaccharides were anthranilic acid (2-AA), labelled as previously described ^28^. Labelled oligosaccharides were purified via mixing with 1ml acetonitrile:H _2_O 97:3 and addition to Discovery DPA-6S columns (pre-equilibrated with 1ml acetonitrile, 2×1ml H _2_O and 2×1ml acetonitrile). The column was washed with 2×1ml acetonitrile:H _2_O 95:5. Purified GSLs were then eluted into 2×0.75ml H _2_O. NP-HPLC was carried out as previously described ^28^, with the following modifications: Solvent A was pure acetonitrile; Solvent B was mQ H _2_O water; Solvent C was 100mM NH _4_OH (pH 3.85) in mQ H _2_O.

### Cholesterol measurement

Cholesterol and cholesterol esters were quantified using an Amplex Red Molecular Probes Kit, according to manufacturer’s instructions. Cellular cholesterol was visualised using filipin (Sigma). Fixed cells were incubated with 1ml filipin working solution (0.05mg/ml in PBS with 0.2% Triton X100) for 1h at room temperature, before being washed with 3×1ml PBS. Imaging was carried out using an Axio Imager A1 microscope in conjunction with an Axiocam High-Resolution Camera and Axiovision software v4.8.

### Cholera toxin B subunit transport assays for GM1 ganglioside trafficking

Cells were washed twice in PBS and incubated with 0.5μM Texas red cholera toxin B subunit (CtxB) for 30min at 37°C followed by a 2h chase in fresh medium at 37°C. Cells were subsequently washed three times with 1% bovine serum albumin in PBS and then fixed in 4% paraformaldehyde. Imaging was carried out using an Axio Imager A1 microscope in conjunction with an Axiocam High-Resolution Camera and Axiovision software v4.8.

### Intracellular sphingomyelin staining

Cells infected with fluorescent live mycobacteria were washed three times with PBS, fixed with paraformaldehyde (4%; 15min) and then stained with the sphingomyelin stain lysenin (0.1μg/ml; Peptides International, Louisville, USA) for 12h at 4°C. The cells were washed with PBS, incubated with lysenin anti-serum (1:500 dilution; Peptide International; rabbit; NLY-14802-v) at 20°C for 1h, and then incubated with a fluorescent secondary antibody (1:200 dilution; donkey anti-rabbit IgG Alexa Fluor 488; Invitrogen Molecule Probes, A21206; RRID: AB_2535792) at 20°C for 30min. Imaging was carried out using an Axio Imager A1 microscope in conjunction with an Axiocam High-Resolution Camera and Axiovision software ver. 4.8.

### LysoTracker staining for fluorescence microscopy

Cells were live stained with 50nM LysoTracker green (Molecular Probes) in PBS at room temperature for 30min prior to washing. Imaging was carried out using an Axio Imager A1 microscope in conjunction with an Axiocam High-Resolution Camera and Axiovision software v4.8.

### Extraction of mycolic acid and fatty acid methyl-esters

Extraction and analysis of total lipids and mycolic acid methyl-esters (MAMES) was carried out with *M. bovis* BCG and genetically modified *M. bovis* BCG, as previously described ^29^. A 100ml culture of bacteria were grown to an absorbance of 1.0 at 600nm, centrifuged (3000g/10min), and the bacteria were resuspended in 5ml PBS [0.137 NaCl, 2.7 KCl, 4.3 Na _2_PO _4_, 1.4 KH _2_PO _4_, pH 7.4 (mM)]. This bacterial solution was transferred to a 8.5ml screw top glass culture tube (VWR International, Lutterworth, UK) and centrifuged (3000g/10min). The supernatant was removed and the bacterial pellet was dried at room temperature overnight under reduced pressure. The desiccated bacterial pellet was incubated with 2ml of 5% aqueous tetrabutylammonium hydroxide at 100°C for 16h. The sample was cooled and 100μl of methyl iodide, 4ml dichloromethane and 2ml H2O was added. The sample was mixed for 30min and the lower organic layer was removed, washed three times with 5ml of H _2_O and dried under nitrogen. The dried extract was resuspended in 1ml diethyl ether, mixed for 60min and centrifuged at 3,000 *x g* for 5min. The supernatant was carefully removed, dried under nitrogen and resuspended in 500μl of dichloromethane to give the MAMES and fatty acid methyl esters (FAMES). The sample was applied to a TLC plate and separated in one dimension with a petroleum ether:acetone (95:5) solvent system. The TLC plate was sprayed with 5% (v/v) molybdophosphoric acid and charred at 110 ^o^C to reveal the lipid species.

### Commercially available mycobacterial lipids

The BCG mycolic fraction and trehalose dimycolate were purchased from Sigma and incubated with cells for the indicated length of time at the indicated concentrations.

### Purification of glycomycolates from mycobacterial cell walls

Dried cell pellets were stirred in 220ml of methanolic saline (20ml of 0.3% NaCl and 200ml of CH _3_OH) and 220ml of petroleum ether for 2h. The biomass was allowed to settle overnight and centrifuged (3000g/5 min). The resulting bi-phasic solution was separated and the upper layer containing non-polar lipids recovered. The lower layer was treated with a further 220ml petroleum ether, mixed and harvested. The two upper petroleum ether fractions were combined and dried under reduced pressure.

To extract polar lipids, a mixture of CHCl _3_/CH _3_OH/NaCl was added to the lower methanolic saline layer. The solution was stirred for 4h and left to settle overnight. This mixture was filtered and the filter cake re-extracted twice with CHCl _3_/CH _3_OH/NaCl solution. Appropriate amounts of CHCl _3_ and NaCl solution were added to the combined filtrates and the mixture stirred for 1h and allowed to settle. The lower layer containing the polar lipids was recovered and dried under reduced pressure. The non-polar and polar lipid extracts were examined by 1D thin-layer chromatography (TLC) on aluminium TLC plates of silica gel 60 F254 (Merck EMD Millipore). Lipids were visualized by spraying plates either with 5% ethanolic molybdophosphoric acid and charring, α-naphthol/sulphuric acid followed by gentle charring of plates for glycolipids, a Dittmer and Lester reagent, which is specific for phospholipids and glycophospholipids, or ninhydrin, an amino-specific reagent for detecting amino residues on extracted lipids.

After analysing the lipid profiles by TLC, purifications were performed using diethylaminoethyl cellulose chromatography. The crude polar lipid extract was dissolved in Solution A [CHCl _3_/CH _3_OH (2:1, v/v)] and a few drops of H _2_O added as necessary to dissolve the lipids. The polar lipid fraction was eluted using Solution A to remove all mycolates, their glycosylated forms and other zwitterionic lipids. Charged lipids were then eluted using ammonium acetate dissolved in Solution A in a stepwise gradient of increasing concentrations of ammonium acetate in C:M ranging from 1mM to 300mM.

The glycolate mycolate fraction was further purified either using silica gel packed into glass columns or by preparative TLC. In the silica gel procedure, the mycolate fraction was dissolved in 100% CHCl _3_ and initially eluted with CHCl _3_/CH _3_OH (80:1, v/v) and further eluted with decreasing concentration of CHCl _3_ [with constant (CH _3_OH)]. The glycomycolate fractions were monitored by TLC on 10×10cm aluminium-backed TLC plates of silica gel 60 F254, and plates developed in either CHCl _3_/CH _3_OH (80:10, v/v) or CHCl _3_/CH _3_OH/H _2_O (65:25:4 v/v/v). The glycomycolates were visualized by spraying with α-naphthol/sulphuric acid followed by gentle charring. In preparative 1D, TLC the mycolate extract was loaded on 10cm × 20cm plastic-backed TLC plates of silica gel 60 F254 (Merck EMD Millipore) and ran in TLC solvent system (CHCl _3_/(CH _3_) _2_CO/CH _3_OH/H _2_O (50:60:2.5:3 v/v/v/v)). TLC plates were subsequently sprayed with either ethanolic Rhodamine 6G (Sigma) for detection of non-polar lipids or 1,6-diphenyl-1,3,5-hexatriene for polar lipids. The lipid bands were visualized, marked under UV light and the corresponding purified lipid spots were scraped from the plates, silica extracted and used for biological testing.

### Quantification of LysoTracker fluorescence via plate reader

Purified glycomycolates were re-suspended in CHCl _3_:EtOH (1:4 v/v) to a concentration of 1mg/ml prior to serial dilution into RPMI to a final concentration of 1ng/ml. A 96-well plate was seeded with RAW 264.7 cells (5×10 ^4^ cells/well), which were allowed to adhere overnight. Glycomycolates were then added prior to 24h incubation at 37 ^o^C/5% CO _2_. Post-incubation, the cells were stained with LysoTracker. Cells were live stained with 50nM LysoTracker green (Molecular Probes) in PBS at room temperature for 30min prior to washing. Fluorescence was quantified using a 96-well plate reader (ex/em, 485/520nm; FLUOstar OPTIMA).

### Visualization of sphingosine in cells

HEK cells were seeded onto 11mm coverslips, placed in wells of a 24-well plate, incubated for 24h and treated with mycobacterial lipids for another 24h. Labelling was performed with a solution of 3µM trifunctional sphingosine (TFS) in imaging buffer (20 HEPES, 115 NaCl, 1.8 CaCl _2_, 1.2 MgCl _2_, 1.2 K _2_HPO _4_ and 0.2% (w/v) glucose (mM)] for 10min. Cells were washed, overlaid with 0.5mL imaging buffer and UV-irradiated on ice for 2.5min at wavelengths >400nm and either immediately crosslinked at wavelengths of >355nm for a further 2.5min, or incubated for 10min at 37°C before crosslinking. Cells were immediately fixed with MeOH at -20°C for 20min. Non-crosslinked lipids were extracted by washing three times with 1mL of CHCl _3_/MeOH/AcOH 10:55:0.75 (v/v) at room temperature. To visualize sphingosine distribution, cells were incubated with 50μl of click mixture [1mM ascorbic acid, 100μM TBTA, 1mM CuSO4 and 2μM Alexa488-azide (Life Technologies) in PBS] for 1h at room temperature in the dark. The coverslips were washed with PBS and mounted onto glass slides using mounting medium. Microscopy images were captured at room temperature using a confocal laser scanning microscope (Zeiss LSM780) with a 63× oil objective (excitation, 488nm; emission, 489–550nm). Images were further processed using Fiji software v1.51g ( http://fiji.sc/Fiji).

### Calcium measurements post-sphingosine uncaging

HeLa cells in 8-well Labteks at 70–80% confluency were labelled with 100μL of 5μM Fluo4 AM solution (Molecular Probes) in imaging buffer [20 HEPES, 115 NaCl, 1.8 CaCl _2_, 1.2 MgCl _2_, 1.2 K _2_HPO _4_ and 0.2% (w/v) glucose (mM)] at 37°C for 30min. In total, 15min prior to the start of the experiment, trifunctional sphingosine (TFS) was added to a final concentration of 2μM. The cells were then washed and kept in imaging buffer at 37°C for the duration of the experiment.

The fluorescence of the calcium indicator Fluo4 was monitored on a dual scanner confocal laser scanning microscope (Olympus FluoView 1200) using a 63× oil objective at 488nm excitation and emission settings between 500–550nm at an interval of 1s per frame. A baseline of 10 frames (= 10s) was captured before photoactivation (‘uncaging’) in a circular region (10 pixel units diameter; 8.9μm ^2^) inside the cells using the tornado function of the Olympus software v3.0. Uncaging was carried out using the 405nm laser line set to 50% intensity for 3s at 2μs per pixel. The time lapse images were analyzed using Fiji software with the FluoQ macro ^30^ set to the following parameters:

- Background subtraction method: Mean of an interactively selected ROI
- Noise reduction/smoothing method: None
- Threshold method: Interactively with ImageJ’s built-in threshold window
- ROI segmentation: Semi-automatically with binary mask modification
- Calculate amplitude changes: Using maximum observed amplitude change

The resulting intensity series/amplitude values represent mean values of whole cells and were loaded in R v3.3.1 ( https://www.r-project.org/) and grouped according to conditions (Ctrl vs. MA vs. TDM). Single cell traces belonging to the same groups were summarized using the R function ‘summarySE’, which calculated the mean, as well as the standard error of the mean, of all traces for every time point. Line and bar graphs were generated using the ggplot2 package ( http://ggplot2.org/) in R v3.3.1.

### Quantification of NPC1/2 levels via western blot

Protein (10μg) was separated on 7% acrylamide gel at 25mA before transfer onto nitrocellulose membrane (Immobilon P (EMD Millipore)) at 40mA/membrane. Membranes were blocked overnight at 4°C in Tris-buffered saline containing 0.1% Tween-20 and 5% powdered milk, before probing with primary antibody against NPC1 (1:5000 dilution; Thermofisher; Rabbit polyclonal; PA1-16187; RRID:AB_2298492) overnight at 4°C. Membrane was then probed with horseradish peroxidase-linked secondary antibody (1:20,000 dilution; Thermo Fisher 31460; goat anti-rabbit polyclonal; RRID: AB_228341) for 1h at room temperature. Membranes were stripped and re-probed with anti-actin specific antibody (1:25,000 dilution; Sigma A3854; mouse monoclonal) for 1h at room temperature to demonstrate equal protein loading into each lane.

### Clearance of *Mycobacterium smegmatis (Msm)*

Host cells grown on coverslips were infected with *Msm* (MOI, 12.5) and incubated at 37°C/5% CO _2_ for 2h. Cells were washed and incubated at 37°C/5% CO _2_ with fresh medium. At stated time points, coverslips were washed, paraformaldehyde fixed and *Msm* clearance quantified via microscopy. Imaging was carried out using an Axio Imager A1 microscope at x63, in conjunction with an Axiocam High-Resolution Camera and Axiovision software v4.8.

### Treatment of infected cells

Cells were infected with BCG 48h prior to washing and addition of the drugs. Cells were fixed (4% paraformaldehyde; 15min at room temperature), and levels of host cell fluorescence (due to the fluorescence of the mCherry-expressing intracellular mycobacteria) quantified by flow cytometry (BD FACS CantoTM II flow cytometer; BD FACSDivaTM software version 6.1; 10,000 events). Curcumin (high purity; Enzo), tetramethylcurcumin (FLLL31; Sigma), cyclodextrin (HPBCD; Sigma) and miglustat (Actelion) were used at the indicated concentrations.

### Assessment of the ability of curcumin analogues to release Ca ^2+^ from the ER

Ca ^2+^ changes in response to curcumin treatment were measured using the genetically encoded O-GECO1 (Addgene plasmid 46025; provided by Robert Campbell) ^31^, since curcumin is fluorescent when incorporated into cells (90% of signal: 370–540nm) and hence precludes the use of standard UV and blue excited Ca ^2+^ dyes. RAW 264.7 macrophages were transfected with 2μg O-GECO1 using jetPRIME (Source Bioscience) and used 24h after transfection. Cells were then incubated with or without 30μM curcuminoids (high purity curcumin; Enzo, FLLL31; Sigma) in tissue culture medium for 1h at 37°C and 5% CO _2_. Recordings were conducted in Ca ^2+^-free medium to eliminate Ca ^2+^ influx. Thus, cells were washed once in a Ca ^2+^-free medium containing (mM): 121 NaCl, 5.4 KCl, 0.8 MgCl _2_, 6 NaHCO _3_, 25 HEPES, 10 glucose, and supplemented with 1mM EGTA and then washed twice in the same medium, except with a lower EGTA concentration (100μM). The cells were mounted on an Olympus IX71 microscope equipped with a 20x UApo/340 objective and a 12-bit Photometrics Coolsnap HQ2 CCD camera. Cells were excited at 543nm using a Cairn monochromator, and emission collected >585nm. Experiments were conducted at room temperature with an image collected every 2s. The effect of the curcuminoids on ER Ca ^2+^ store depletion was tested by subsequent addition of 2μM ionomycin (Sigma), which releases Ca ^2+^ from the ER in control cells. At the end of each run, 10mM CaCl _2_ was added to verify O-GECO1 expression and viability of the cells. Images were analysed on a single-cell basis using Optafluor software v7.6.3.0 and Microsoft Excel 2013. The fluorescence of high-purity curcumin (815 ± 35RFU) was subtracted from the O-GECO1 signal.

### Effect of calcium chelation on curcumin efficacy

RAW 264.7 cells were infected with FLUOS-labelled *M.bovis* BCG and incubated at 37°C for 6h. Cells loaded with BAPTA-AM (Sigma) and were incubated with this substrate at 20μM for 30min before the addition of curcumin. Following incubation, the cells were washed three times with PBS, fixed with 4% paraformaldehyde and stained with Filipin.

### Assessment of the effect of curcuminoids on BCG growth in broth

Exponentially growing BCG culture in 7H9 (20ml containing ∼5×10 ^8^ cells/ml) was diluted into 100ml in the presence of 30μM curcuminoids. Growth was measured spectrophotometrically (Jenway 6305 spectrophotometer) via absorbance at 600nm.

### Statistical analysis

All statistical analysis was performed with Graphpad Prism 6.

## Results

### Infection with pathogenic mycobacteria induces NPC phenotypes in murine and human macrophages

NPC cells display a unique combination of phenotypes, including reduced LE/Lys Ca ^2+^ levels ^21, 22^, and mistrafficking and storage of sphingosine, glycosphingolipids (GSLs), cholesterol and sphingomyelin ^32^. Induction of these phenotypes in wild-type cells post-infection with intracellular mycobacteria would therefore be indicative of NPC pathway inhibition. We infected RAW 264.7 murine macrophages with live BCG (Pasteur strain), an attenuated form of *M. bovis,* which is commonly used to model early stage *Mtb* infection. To assess the effect of infection on lysosomal Ca ^2+^, we first monitored Ca ^2+^ content indirectly by releasing Ca ^2+^ from the lumen to the cytosol with the lysomotropic agent glycyl-L-phenylalanine-β-napthylamide (GPN). We have previously shown that GPN responses faithfully reflect lysosomal Ca ^2+^ levels ^22^.

**Figure 1.**
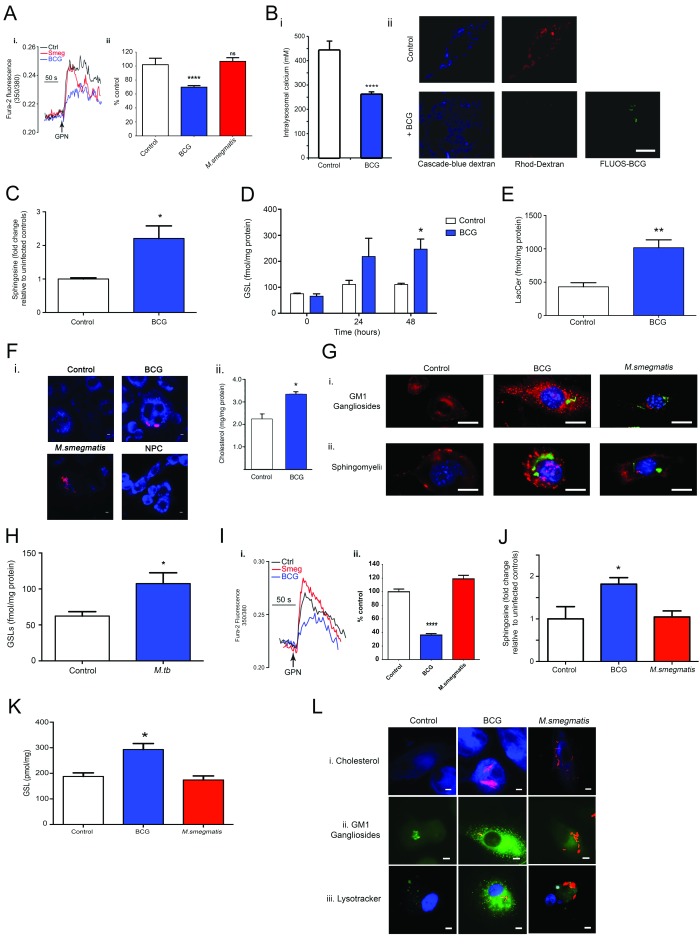
Pathogenic mycobacteria induce NPC phenotypes in RAW 264.7 cells and human macrophages. **(A)** Lysosomal Ca ^2+^ levels in mycobacterial-infected RAW 264.7 macrophages as quantified by measuring GPN-induced release of lysosomal Ca ^2+^ (24h infection; MOI, 12.5). (i) Ca ^2+^ responses from representative single fura-2 loaded RAW 264.7 cells upon addition of GPN (point of addition indicated by arrow). At the end of each run all cells responded to 1μM ionomycin. (ii) Maximal Ca ^2+^ response upon addition of GPN as determined by the difference between basal and maximum fura-2-ratio (Δ350/380). Changes given as percentage difference relative to Δ350/380 in uninfected control. Mean ± SEM of n=167–311 individual cells per group. **** *p*<0.001 vs uninfected control (via 1-way ANOVA). **(B)** Intra-lysosomal [Ca ^2+^] in BCG-infected RAW 264.7 cells quantified by loading cells with low-affinity Rhod-dextran and Cascade blue-dextran (18h infection; MOI, 12.5). (i) Mean ± SEM of intralysosomal [Ca ^2+^] in 90 cells/group following BCG infection (dextran). **** *p*<0.001 vs uninfected control (student t-test) (ii) Representative images of dextran-loaded cells. Scale bar, 5μm. **(C)** Sphingosine levels in BCG-infected RAW 264.7 macrophages (48h infection; MOI, 12.5). Values adjusted for sample protein concentration. Mean ± SEM. N=4. * *p*<0.05 vs uninfected control (student t-test). **(D)** GSL levels in BCG-infected RAW 264.7 macrophages (MOI, 12.5). Values adjusted for sample protein concentration. Mean ± SEM. N=4. * *p*<0.05 vs uninfected control (student t-test). **(E)** LacCer levels in BCG-infected RAW 264.7 macrophages (1 week infection; MOI, 12.5). Values adjusted for sample protein concentration. Mean ± SEM. N=4. * *p*<0.05 vs uninfected control (student t-test). **(F)** (i) Cholesterol distribution in mycobacteria-infected RAW 264.7 macrophages (24h infection; MOI, 12.5) or in RAW 264.7 macrophages with a U18666A-induced NPC phenotype (48h treatment, 2μg/ml). Blue, filipin (cholesterol); red, mCherry-expressing mycobacteria. Scale bar, 5μm (ii) Quantification of cholesterol storage in BCG-infected RAW 264.7 macrophages (18h infection; MOI 12.5). Values are adjusted for sample protein concentration. Mean ± SD. N=3. * *p*<0.05 vs uninfected control (student t-test). **(G)** (i) Trafficking of GM1 ganglioside in mycobacteria-infected RAW 264.7 macrophages (18h infection; MOI, 12.5). Green, FLUOS-labelled mycobacteria; red, cholera toxin subunit B (GM1 ganglioside); blue, Hoescht 33258 (nucleus). (ii) Sphingomyelin distribution in mycobacteria-infected RAW 264.7 macrophages (18h infection; MOI, 12.5). Green, FLUOS-labelled mycobacteria; red, lysenin (sphingomyelin); blue, Hoescht 33258 (nucleus). Scale bar, 5μm. **(H)** GSL levels in *Mtb*-infected RAW 264.7 macrophages. (MOI 12.5) Values adjusted for sample protein concentration. Mean ± SEM. N=4. * *p*<0.05 vs uninfected control (student t-test). **(I)** Lysosomal Ca ^2+^ levels in mycobacterial-infected primary human macrophages as quantified by GPN-induced release of lysosomal Ca ^2+^ (24h infection; MOI, 12.5) (i) Ca ^2+^ responses from representative single fura-2 loaded primary human macrophages upon addition of GPN (point of addition indicated by arrow). At the end of each run all cells responded to 1μM ionomycin. (ii) Maximal Ca ^2+^ response upon addition of GPN as determined by the difference between basal and maximum fura-2-ratio (Δ350/380). Changes are given as percentage difference relative to Δ350/380 in uninfected control. Mean ± SEM of n=71–173 individual cells per group. **** *p*<0.001 vs uninfected control (1-way ANOVA). **(J)** Sphingosine levels in mycobacteria-infected primary human macrophages (48h infection; MOI, 12.5) Values adjusted for sample protein concentration. Mean ± SEM. N=4. * *p*<0.05 vs uninfected control (1-way ANOVA). **(K)** GSL levels in mycobacteria-infected primary human macrophages (48h infection; MOI, 12.5). Values adjusted for sample protein concentration. Mean ± SEM. N=4. * *p*<0.05 vs uninfected control (1-way ANOVA). **(L)** (i) Cholesterol distribution in mycobacteria-infected primary human macrophages. Blue, filipin (cholesterol); red, mCherry-expressing mycobacteria. (ii) Trafficking of GM1 ganglioside in mycobacteria-infected primary human macrophages. Green, cholera toxin subunit B (GM1 ganglioside); red, mCherry-expressing mycobacteria. (iii) Lysosomal expansion in mycobacteria-infected primary human macrophages. Green, LysoTracker (LE/Lys); red, mCherry-expressing mycobacteria; blue, Hoescht 33258 (nucleus) (24h infection; MOI, 12.5). Scale bar, 5μm. NPC, Niemann-Pick Type C disease; GPN, glycyl-L-phenylalanine-β-napthylamide; BCG, Bacillus Calmette-Guerin; GSL, glycosphingolipid; LacCer, lactosylceramide.

In agreement with known NPC cellular phenotypes ^22^, BCG-infected macrophages exhibited a significant decrease in LE/Lys-mediated Ca ^2+^ release compared to the uninfected population ( Figure 1A; ***p*<0.001**), consistent with less Ca ^2+^ within the lysosomes of BCG-infected macrophages. In contrast, infection with the environmental mycobacteria *M. smegmatis (Msm)* gave no significant change in GPN responses ( Figure 1A). The significant decrease in the GPN response with BCG could not simply be accounted for by changes in basal cytosolic Ca ^2+^ ( Supplementary Figure 1) nor by changes in the activity of the lysosomal enzyme cathepsin C, which is responsible for hydrolysing GPN and thereby inducing lysosomal osmotic stress and Ca ^2+^ release ( Supplementary Figure 2). Consistent with results using the indirect approach, direct measurement of endo-lysosomal Ca ^2+^ content with a luminal Ca ^2+^-dye (low-affinity Rhod-dextran) confirmed reduced levels of lysosomal Ca ^2+^ in BCG-infected RAW cells ( Figure 1B; ***p*<0.001**). As in NPC cells, macrophages infected with BCG exhibited a significant accumulation of sphingosine ( Figure 1C; ***p*<0.05**) and glycosphingolipids ( Figure 1D; ***p*<0.05**). Accumulation of lactosylceramide (LacCer) (the levels of which are elevated in NPC cells/ tissues of *Npc1* ^-/-^ mice and in the caseum from human TB granulomas ^33^) was not detected at 24 and 48h post-infection (BCG-infected RAW 264.7 cells), but was significantly elevated 7 days post-infection ( Figure 1E; ***p*<0.01**). The most widely recognised cellular hallmark of NPC cells is the storage of cholesterol within LE/Lys ^18, 34^, detected using the fluorescent cholesterol-binding antibiotic filipin. Cholesterol accumulation similar to that observed in NPC cells was observed in punctate structures in BCG-infected RAW 264.7 cells, but not in cells infected with non-pathogenic *Msm* ( Figure 1Fi). Biochemical quantitation of cholesterol confirmed higher levels in BCG-infected cells ( Figure 1Fii; ***p*<0.05)**. The fold-change increase in levels of lipids (glycosphingolipids, sphingosine and cholesterol) and the reduction in lysosomal calcium release observed in wild-type macrophages infected with BCG was comparable to that observed in both *Npc1* mutant cells and cells in which a NPC disease phenotypes have been pharmacologically induced ^22^. Interestingly, storage of cholesterol was not restricted to cells infected with BCG; neighbouring, uninfected cells also displayed elevated cholesterol storage ( Figure 1Fi), suggesting that local paracrine factors capable of inducing NPC phenotypes are released from infected cells. Other cellular hallmarks of NPC, such as sphingomyelin and GSL accumulation, were also induced by BCG infection, but not by *Msm*. This was demonstrated using fluorescently conjugated cholera toxin subunit B and lysenin that measure the storage and mislocalisation of GM1 ganglioside ( Figure 1Gi) and sphingomyelin respectively ( Figure 1Gii). To determine the relevance of our findings with BCG to *Mtb*, we infected the same cell line with live *Mtb* (H37Rv strain). Total cellular GSLs were significantly elevated 48h post *Mtb* infection ( Figure 1H; ***p*<0.05).**

**Figure 2.**
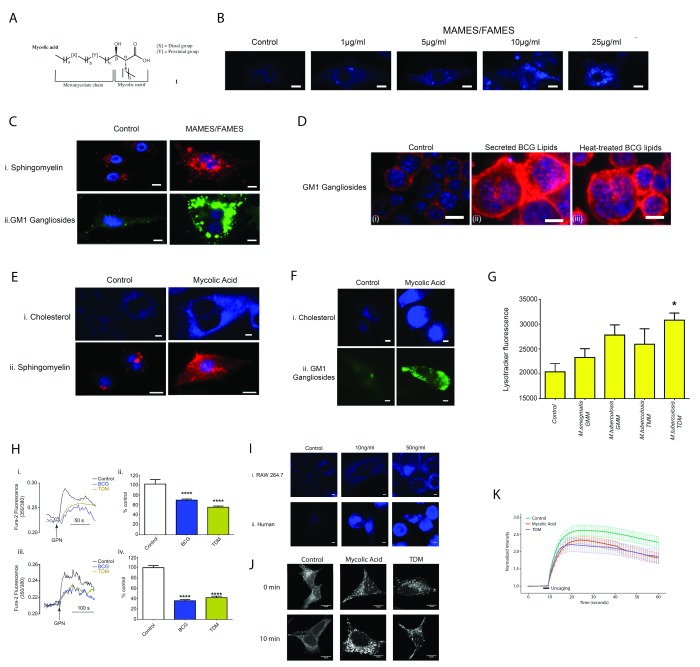
Mycobacterial cell wall lipids induce NPC phenotypes in the absence of live mycobacteria. **(A)** General structure of mycolic acids. The lipid consists of a mycolic motif (with alkyl chain of variable length) and meromycolate chain with a distal and proximal functional group (X/Y) (adapted from 74). **(B)** Cholesterol storage in RAW 264.7 macrophages treated with BCG MAMES/FAMES (18h treatment). Blue, filipin (cholesterol). Scale bar, 5μm. **(C)** (i) Trafficking of GM1 ganglioside in RAW 264.7 macrophages treated with MAMES/FAMES. Red, cholera toxin subunit B (GM1 ganglioside); blue, Hoescht 33258 (nucleus). (ii) Sphingomyelin distribution in RAW 264.7 macrophages treated with MAMES/FAMES. Green, lysenin (sphingomyelin); blue, Hoescht 33258 (nucleus). (MOI 12.5; 5μg/ml). Scale bar, 5μm. **(D)** Trafficking of GM1 ganglioside in (i) untreated RAW 264.7 macrophages, post 18h incubation with secreted BCG lipids (ii) or heat-treated lipids (iii). Red, cholera toxin subunit B (GM1 gangliosides); blue, Hoescht 33258 (nucleus). **(E)** (i) Cholesterol storage in RAW 264.7 macrophages treated with BCG mycolic acids. Blue, filipin (cholesterol); red, propidium iodide (nucleus). (ii) Sphingomyelin distribution in RAW 264.7 macrophages treated with BCG mycolic acids. Red, lysenin (sphingomyelin); blue Hoescht 33258 (nucleus) (24h treatment; 5μg/ml). Scale bar, 5μm. **(F)** (i) Cholesterol storage in primary human macrophages treated with BCG mycolic acids. Blue, filipin (cholesterol) (ii) Trafficking of GM1 ganglioside in primary human macrophages treated with BCG mycolic acids. Green, cholera toxin subunit B (GM1 ganglioside) (24h treatment; 5μg/ml). Scale bar, 5μm. **(G)** LysoTracker staining of RAW 264.7 macrophages 24h post incubation with purified mycolic acid esters (glycomycolates; 1ng/ml). Mean ± SEM. N=4. * *p*<0.05 vs untreated control (1-way ANOVA). **(H)** Lysosomal Ca ^2+^ levels in RAW 264.7 (i, ii) and primary human macrophages (iii, iv) treated with commercial BCG TDM as quantified by GPN-induced release of lysosomal Ca ^2+^ (24h treatment; 50ng/ml). (i, iii) Ca ^2+^ responses from representative single fura-2 loaded RAW 264.7/primary human macrophages upon addition of GPN (point of addition indicated by arrow). (ii, iv) Maximal Ca ^2+^ response upon addition of GPN as determined by the difference between basal and maximum fura-2-ratio (Δ350/380). Changes given as percentage difference relative to Δ350/380 in uninfected control. Mean ± SEM of n=127–252 (RAW 265.7) and 71–156 (human) individual cells per group. **** *p*<0.001 vs untreated control (1-way ANOVA). **(I)** Cholesterol storage in RAW 264.7 (i) and primary human macrophages (ii) treated with BCG TDM. Blue, filipin (cholesterol) (50ng/ml; 24h treatment). Scale bar, 5μm. **(J)** Subcellular localization of sphingosine in HEK293 cells treated with either BCG MA (5μg/ml) or BCG TDM (50ng/ml). Cells were treated with lipids/glycomycolates for 24h prior to investigation of sphingosine localization. Cells were incubated with 3μM trifunctional sphingosine for 10min prior to washing and either immediately subjected to photo-crosslinking and MeOH fixation (0min) or incubated for 10min before crosslinking/fixation (10min). Visualization achieved by clicking Alexa488-azide to terminal alkyne bond of sphingosine. Scale bar, 10μm. **(K)** Sphingosine-induced calcium release from HeLa cells pre-treated with either BCG MA (5μg/ml) or BCG TDM (50ng/ml). Cells were treated with lipids/glycomycolates for 24h prior to investigation of sphingosine-induced calcium release. Mean Fluo-4 fluorescence of control and lipid/glycomycolate-treated HeLa cells upon UV-induced uncaging of trifunctional sphingosine (point of uncaging indicated). Traces represent mean values of 13–21 cells per group, with the standard error of the mean plotted as error bars. GPN, glycyl-L-phenylalanine-β-napthylamide; MAMES/FAMES, mycolic acid methylesters / fatty acid methyl esters.

To determine whether our findings in a murine macrophage cell line would be replicated in primary human macrophages, which are more relevant for *Mtb* infection/TB, monocyte-derived macrophages from healthy donors were infected with BCG and *Msm*. We observed that BCG infection was associated with reduced LE/Lys-mediated Ca ^2+^ release ( Figure 1I; ***p<*0.001)**, increased levels of sphingosine ( Figure 1J; ***p<*0.05)** and elevated GSLs ( Figure 1K; ***p<*0.05)**. Cholesterol storage in LE/Lys was also detected in BCG-infected human macrophages and in non-infected neighbouring cells ( Figure 1Li), accompanied by mistrafficking of GM1 ganglioside ( Figure 1Lii). Significant expansion of the lysosomal compartment, as visualised with LysoTracker (another hallmark of lysosomal storage disorders, including NPC) ^35, 36^), was also detected ( Figure 1Liii). None of these changes occurred in human macrophages infected with non-pathogenic *Msm* ( Figure 1Li–iii). Electron microscopy revealed that BCG-infected cells showed both the presence of intracellular mycobacteria and electron-dense lamellar storage bodies. These were similar to those observed in uninfected Kupffer cells in the liver of *Npc1 ^-/-^* mice and in cells with pharmacologically-induced NPC phenotypes (U18666A treatment) ( Supplementary Figure 3). In contrast, cells infected with *Msm* exhibited no evidence of storage bodies. Together, these data indicate that pathogenic mycobacteria induce cellular phenotypes indistinguishable from the lysosomal storage disease, NPC.

### Mycobacterial cell wall lipids induce NPC phenotypes

Cholesterol accumulation was observed in non-infected as well as infected cells ( Figure 1F and Li). We hypothesised that there is a factor(s) derived from BCG and *Mtb* that inhibits the NPC pathway of the host cells and that is also released from infected cells and endocytosed by non-infected neighbouring cells, wherein it also induces NPC pathway dysfunction.

It has previously been shown that mycolic acids (a group of long chain β-hydroxy fatty acids that constitute a major component of the mycobacterial cell wall ( Figure 2A) may play a role in enabling the intracellular persistence of some mycobacterial species ^37^. Whilst mycolic acids are present in the cell walls of both intracellular and environmental mycobacteria, there are certain structural features present in the mycolic acids found in those species capable of persisting within host cells, such as increased levels of cyclopropanation ^38^.

A purified lipid fraction consisting of mycolic acid methyl esters (MAMES) and fatty acid methyl esters (FAMES) from the cell wall of BCG was applied to wild-type murine macrophages. We observed that BCG MAMES/FAMES induced accumulation/re-distribution of cholesterol in a dose-dependent manner ( Figure 2B). MAMES/FAMES treatment also induced mistrafficking of GM1 ganglioside ( Figure 2Ci) and accumulation/re-distribution of sphingomyelin ( Figure 2Cii), similar to that observed in both NPC cells and wild-type RAW 264.7 macrophages infected with live BCG ( Figure 1G). Heat-treating the MAMES/FAMES mixture did not affect the mixture’s ability to affect GM1 ganglioside distribution, suggesting that the NPC phenotype-inducing factor was a lipid ( Figure 2D). Further experiments with a commercially available mycolic acid fraction from the BCG cell wall supported the role of this lipid class in inducing NPC phenotypes, as this fraction induced accumulation of cholesterol and GM1 gangliosides in both wild-type RAW 264.7 macrophages ( Figure 2E) and primary human macrophages from healthy donors ( Figure 2F).

Within the mycobacterial cell wall mycolic acids may be present as free lipid or esterified to sugars to form glycomycolates. Note that the name of a glycomycolate indicates the identity of the sugar molecule and the number of mycolic acid motifs to which it is esterified. One such glycomycolate from *Mtb*, trehalose dimycolate (TDM) (consisting of two mycolic acid motifs esterified to a trehalose sugar), has previously been shown to prevent phagosomal maturation and induce formation of caseating granulomas and foamy macrophages in the absence of the mycobacteria itself ^39– 41^. We assayed the effect of purified glycomycolates obtained from both intracellular and environmental mycobacteria on LysoTracker fluorescence (reflecting relative lysosomal volume). Treatment with *Mtb* TDM was associated with a significant increase in LysoTracker fluorescence, indicative of lysosomal storage ( Figure 2G; ***p*<0.05**). Glucose monomycolate (GMM) and trehalose monomycolate (TMM) from *Mtb* caused only modest lysosomal expansion, whilst GMM from *Msm* had a minimal effect. Crucially, commercially available TDM from BCG ** induced NPC phenotypes in the absence of the bacteria itself, including reduced LE/Lys-mediated Ca ^2+^ release ( Figure 2H; ***p<*0.001**) and accumulation of cholesterol ( Figure 2I) in both murine and human macrophages. The reduction in LE/Lys-mediated Ca ^2+^ release post-TDM treatment was comparable to that induced by BCG itself ( Figure 2H). Both TDM and MA were also observed to have a deleterious effect on the ability of cells to traffic sphingosine. These experiments utilised a novel, trifunctional sphingosine probe ^42^, in which the lipid is covalently attached to a photolabile group, rendering it biologically inactive. Whilst the caged form is taken up into cells, it is not metabolised. Upon exposure to UV light the biologically active form of the lipid is released within cells ^42^. In addition to the photolabile group, the trifunctional sphingosine used in this experiment also features a diazirine moiety, enabling photo-activated crosslinking, and a functionality that allows the sphingosine to be fluorescently labelled post-fixation. HEK293 cells were subjected to a 10min pulse with trifunctional sphingosine. Immediately post-uncaging, sphingosine was localized to the late-endosome/lysosome in both the control and lipid/glycomycolate-treated cells (0min) ( Figure 2J). After a 10min chase period post-uncaging, the punctate sphingosine localization pattern was much less pronounced in control cells, indicating movement of the lipid out of the lysosome. This movement was much less pronounced in the MA/TDM-treated cells, as indicated by the sphingosine sequestration to the punctate structures of the LE/Lys, as previously shown for NPC-patient fibroblasts ^36^. A sudden increase in intracellular sphingosine, as achieved by uncaging, was previously demonstrated to induce a transient rise in cytosolic calcium mediated by the lysosomal TPC1 calcium channel ^42^. Upon sphingosine uncaging, calcium transients were reduced in MA/TDM-treated HeLa cells, relative to untreated controls ( Figure 2K). This is in agreement with the experiments shown above, in which the amount of calcium released by GPN treatment was significantly reduced as a result of TDM treatment ( Figure 2H).

### Mycobacteria target the NPC1 protein

**Figure 3.**
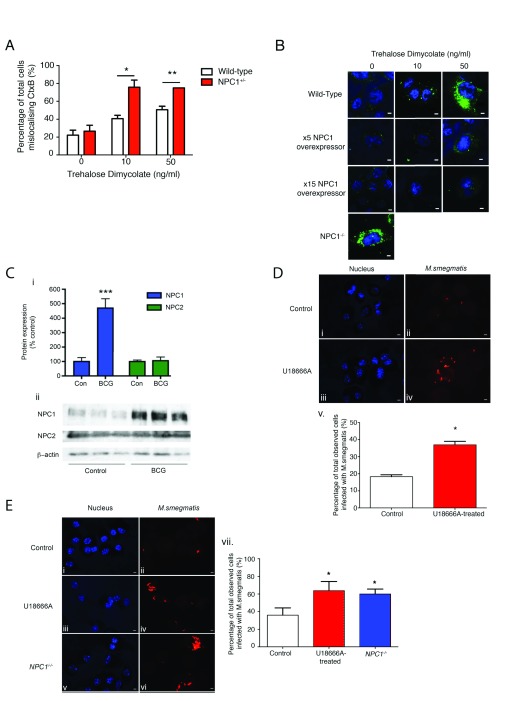
Pathogenic mycobacteria inhibit NPC1. **(A)** Localisation of GM1 ganglioside in bone marrow macrophages from wild-type and *Npc1 ^+/-^* mice treated with purified *Mtb* TDM (48h treatment). Expressed as percentage of total number of cells mislocalising GM1. Mean ± SEM. N>40 cells/group. * *p*<0.05; ** *p*<0.01 vs treated wild-type (1-way ANOVA). **(B)** Trafficking of GM1 ganglioside in CHO cells expressing variable levels of NPC1 protein post-treatment with commercial BCG TDM. Green, cholera toxin subunit B (GM1 ganglioside); blue, Hoescht 33258 (nucleus) (48h treatment). Scale bar, 5μm. **(C)** (i.) Quantification of NPC1/NPC2 protein levels in BCG-infected RAW 264.7 macrophages, as determined by western blot (48h infection; MOI, 12.5). Mean ± SEM. N=3. *** *p*<0.01 vs control (student t-test) (ii) Western blot showing NPC1 and NPC2 bands and loading control β-actin. **(D)** (i–iv) Persistence of *M. smegmatis* in untreated (i and ii) primary human macrophages or macrophages pre-treated with U18666A (iii and iv) at 2μg/ml for 48h prior to 2h infection (MOI, 12.5), washing and 18h incubation. Blue, Hoescht 33258 (nucleus); red, mCherry-expressing *M. smegmatis* (v) Quantification of results shown in upper panel. Mean ± SEM. N>84 individual cells per group. * *p*<0.05 vs untreated control (student t-test). **(E)** (i–vi) Persistence of *M. smegmatis* in untreated (i, ii) and U18666A-treated (iii ,iv) resident peritoneal macrophages from wild-type mice and from untreated resident peritoneal macrophages from *Npc1 ^-/-^* mice (v,vi). Treatment with U18666A at 2μg/ml for 48h prior to 2h infection with *M. smegmatis* (MOI, 12.5), washing and 4h incubation. Blue, Hoescht 33258 (nucleus); red, mCherry-expressing *M. smegmatis* (vii) Quantification of results shown in left panel. Mean ± SEM. N>73 individual cells per group. * *p*<0.05 vs untreated wild-type control (1-way ANOVA). CHO, Chinese hamster ovary cells; BCG, Bacillus Calmette-Guerin; TDM, trehalose dimycolate.

Inhibition of the host NPC pathway could occur at the level of the NPC1 or NPC2 protein. Mutations in either the *NPC1* or *NPC2* genes gives identical cellular phenotypes ^43^. If TDM inhibited the NPC pathway via interaction with NPC1, we reasoned that heterozygous NPC1 cells would be more susceptible to inhibition than wild-type cells due to reduced NPC1 protein levels. In the absence of TDM the proportion of cells with mislocalised GM1 was not significantly different between populations of ** bone marrow-derived macrophages generated from wild-type and *Npc1* ^+/-^ mice ( Figure 3A; ***p*>0.05)**. Incubation of bone marrow-derived macrophages with TDM revealed that macrophages from *Npc1 ^+/-^* mice were more susceptible to glycomycolate-induced lipid mislocalization relative to their wild-type counterparts, with a given concentration of TDM causing a great percentage of the heterozygous cells to mislocalise GM1 ganglioside ( Figure 3A; ***p<*0.05/0.01)**. Conversely, CHO cells overexpressing NPC1 were more resistant to TDM-induced NPC cellular phenotypes than wild-type cells. Whereas wild-type cells incubated with 50ng/ml TDM exhibited dramatic mistrafficking of GM1, the effects were much less pronounced in the overexpressing cells. Cells overexpressing NPC1 by 15-fold were more resistant than those over-expressing NPC1 5-fold ( Figure 3B). We examined NPC1 and NPC2 protein expression levels in RAW 264.7 cells infected with BCG. NPC1 was significantly upregulated in infected cells ( Figure 3C; ***p<*0.001)**, with no changes in NPC2 levels.

Mycobacterial species, such as *Msm,* are readily cleared by healthy cells, due to their inability to inhibit phagosome-lysosome fusion. One prediction arising from the above experiments is that a pre-existing dysfunction in the NPC pathway and subsequent defects in lysosomal fusion (as found in NPC patient cells) will render a cell less able to clear typically non-intracellular mycobacteria. Consistent with this hypothesis, RAW 264.7 cells, in which an NPC phenotype was induced by treatment with U18666A (a widely-used pharmacological inducer of NPC phenotypes in wild-type cells that targets NPC1) ^44^, had an impaired ability to clear non-pathogenic *Msm* ( Figure 3D; ***p<*0.05**) relative to untreated RAW 264.7 macrophages. Impaired clearance of *Msm* was also observed in *Npc1 ^-/-^* and U18666A-treated wild-type bone marrow-derived mouse macrophages, ( Figure 3E; ***p<*0.05**).

### NPC therapeutics promote clearance of pathogenic mycobacteria

**Figure 4.**
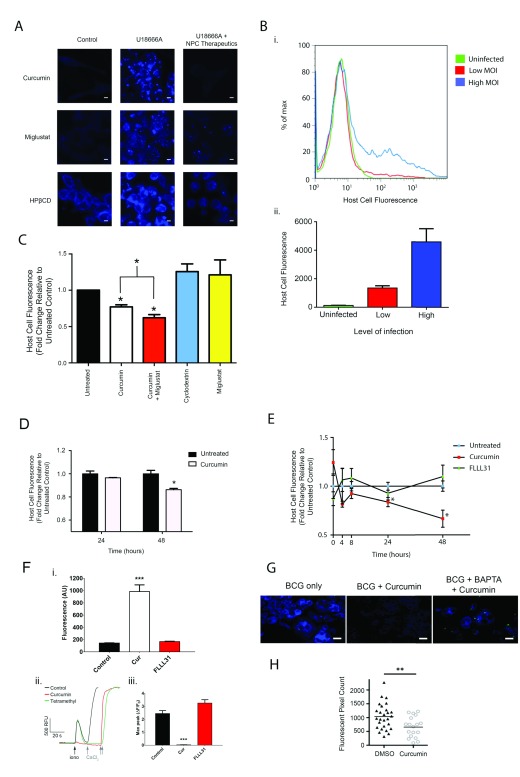
Certain NPC therapeutics promote clearance of intracellular mycobacteria. **(A)** Cholesterol distribution in wild-type RAW 264.7 macrophages treated with U18666A (2μg/ml) for 48h and subsequently treated with either vehicle (DMSO), curcumin (30μM/24h), miglustat (50μM/72h) or hydroxypropyl-β-cyclodextrin (250μM/24h). Blue, filipin (cholesterol). Scale bar, 5μm. **(B)** Correlation between extent of infection with mCherry-expressing BCG and levels of RAW 264.7 fluorescence as quantified using flow cytometry (i) Representative histograms showing fluorescence of RAW 264.7 cell cultures infected with BCG at low or high MOI (10 or 100 respectively). (ii) Fluorescence of RAW 264.7 cell cultures infected with BCG at low or high MOI (10,000 cells counted). Mean ± SEM, N=4. **(C)** Effect of curcumin on intracellular BCG levels in RAW 264.7 macrophages. Fold change in mean fluorescence of RAW 264.7 macrophages after 48h infection with mCherry-BCG (MOI, 12.5) and subsequent treatment with curcumin (30μM; 24h), miglustat (50μM; 72h), combined curcumin (30μM; 24h) and miglustat (50μM; 72h) or hydroxypropyl-β-cyclodextrin (250μM; 24h). Fold change in fluorescence given relative to untreated, infected control. Mean ± SEM. N=4. * *p*<0.05 vs untreated, infected control (1-way ANOVA). **(D)** Effect of curcumin on intracellular BCG levels in primary human macrophages. Fold change in mean fluorescence of primary human macrophages after 48h infection with mCherry-BCG (MOI, 12.5) and subsequent treatment with 30μM curcumin. Fold change in fluorescence given relative to untreated, infected controls. Mean ± SEM. N=4. * *p*<0.05 vs untreated, infected control (1-way ANOVA). **(E)** Effect of curcuminoids on intracellular BCG levels in RAW 264.7 macrophages. Fold change in mean fluorescence of RAW 264.7 macrophages after 48h infection with mCherry-BCG (MOI, 12.5) and subsequent 24h treatment with 30μM curcumin or curcumin analogue FLLL31. Fold change in fluorescence given relative to untreated, infected controls. Mean ± SEM. N=4. * *p*<0.05 vs untreated, infected control (1-way ANOVA). **(F)** Effect of curcuminoids on ER Ca ^2+^ store depletion in uninfected RAW 264.7 macrophages. RAW 264.7 macrophages were transfected with the fluorescent Ca ^2+^ reporter O-GECO1 ^31^ (i) Raw fluorescence (arbitrary units, AU) in RAW 264.7 macrophages post-treatment with curcuminoids (30μM/1h). Mean ± SEM of n=42–125 individual cells per group. *** *p*<0.0001 vs untreated control (1-way ANOVA) (ii) Representative single-cell Ca ^2+^ traces of RAW 264.7 macrophages pre-treated with curcumin or FLLL31 normalised to the “basal” (min) (i) and Ca ^2+^-induced (max) dynamic range; 2µM ionomycin and 10mM CaCl _2_ were added when indicated by the arrows. (iii) Maximum Ca ^2+^ responses (ΔF/ F _0_) upon addition of 2μM ionomycin. Mean ± SEM of n= 42–105 individual cells per group. **** *p*<0.0001 vs untreated control (1-way ANOVA). **(G)** Effect of intracellular calcium chelation on beneficial effects of curcumin. RAW 264.7 macrophages were infected with FLUOS-labelled BCG (18h; MOI, 12.5) prior to treatment with either curcumin alone (6h/10μM) or BAPTA-AM (30min/20μM) prior to addition of curcumin (6h/10μM). Blue, filipin (cholesterol); green, FLUOS-labelled BCG. Scale bar, 5μm. **(H)** Effect of curcumin on *M. marinum* burden in infected zebrafish larvae. Larvae were infected for 2 days post fertilisation and subsequently treated for 48h with vehicle (DMSO) or curcumin (1.5μM). Fluorescent pixel count is a measure of the overall bacterial burden in the larvae. ** *p*<0.01 vs DMSO-treated control (1-way ANOVA).

A number of compounds correct NPC cellular phenotypes. These include curcumin (a modulator of intracellular Ca ^2+^ ^22^), miglustat (an imino sugar inhibitor of GSL biosynthesis that is EMA-approved for NPC therapy ^45, 46^) and β-cyclodextrin (HPβCD; a cyclic oligosaccharide efficacious in animal models of NPC ^47– 50^). All three compounds are capable of reducing levels of cholesterol storage in genetically and pharmacologically induced NPC cells ( Figure 4A). Infection with intracellular mycobacteria induces phenotypes associated with NPC in wild-type cells. Those compounds capable of correcting NPC phenotypes were therefore investigated for any effect on promoting clearance of intracellular mycobacteria from infected host macrophages. The concentrations and duration of treatments used in these clearance experiments ( Figure 4B–E) were identical to those demonstrated to correct U18666A-induced NPC cellular phenotypes ( Figure 4A). Flow cytometry was used to determine the extent to which host cells were infected with fluorescent BCG, with increasing MOIs associated with increased host cell fluorescence ( Figure 4B). RAW 264.7 cells were infected with mCherry-expressing BCG for 48h (MOI 12.5, as per Figure 1) then treated with NPC-correcting compounds. A decrease in host cell fluorescence was indicative of reduced levels of intracellular mCherry-expressing BCG. Treatment with curcumin was associated with significantly lower levels of host cell fluorescence (potentially reflecting enhanced clearance) relative to untreated cells ( Figure 4C; ***p*<0.05)**. Miglustat and cyclodextrin had no significant benefit, although combining miglustat and curcumin showed a small but significant benefit relative to curcumin alone ( Figure 4C; ***p*<0.05)**. Curcumin also significantly reduced host cell fluorescence in infected primary human macrophages ( Figure 4D; ***p*<0.05**).

Curcumin is hypothesized to be beneficial in NPC cells due to its inhibition of the sarco-endoplasmic reticulum Ca ^2+^-ATPase (SERCA) ^51^. This inhibition leads to decreased Ca ^2+^ re-uptake into the ER, so that cytosolic Ca ^2+^ levels remain elevated for longer. The increased availability of Ca ^2+^ within the cytosol is able to at least partially compensate for the reduced lysosomal Ca ^2+^ release seen in NPC cells, and overcome the block in LE/Lys fusion ^22^. The enhancement of BCG clearance by curcumin was dependent upon its ER Ca ^2+^-mobilising properties. This was assessed in two ways: we first tested the ability of curcuminoids to increase cytosolic Ca ^2+^ and subsequently assessed whether this Ca ^2+^ emanated from the ER by probing residual ER Ca ^2+^ store content with ionomycin which, under these conditions, targets the ER Ca ^2+^ stores. The ability of a curcuminoid to reduce mycobacterial load correlated with its ability to modulate host cell Ca ^2+^. A curcumin analogue FLLL31 (tetramethylcurcumin) had no effect on either host cell fluorescence (indicative of intracellular BCG levels) ( Figure 4E) or host cell cytosolic Ca ^2+^ and ER Ca ^2+^ levels assessed with ionomycin ( Figure 4F; ***p*<0.001)**. In contrast, curcumin, which reduced host cell fluorescence, did increase cytosolic Ca ^2+^ via mobilization of the ER Ca ^2+^ stores ( Figure 4F; ***p*<0.001**). The importance of host cell Ca ^2+^ in promoting BCG clearance is further supported by loading the cytosol with the Ca ^2+^ chelator BAPTA. Co-incubating infected cells with curcumin and membrane-permeant BAPTA/AM abrogates the beneficial effect of curcumin on both host cell fluorescence/mycobacterial burden and levels of host cell cholesterol ( Figure 4G). Note that whilst curcuminoids have direct anti-BCG activity in host-cell free systems ( Supplementary Figure 4) the kinetics of this anti-bacterial action are too slow to account for the relatively rapid effects we observed: it took >4 days for curcumin to reduce BCG growth in broth. The evidence presented here supports a model in which curcumin promotes mycobacterial clearance by providing an alternative source of Ca ^2+^ that can compensate for the reduced lysosome-mediated Ca ^2+^ release observed in host cells infected with intracellular mycobacteria ( Figure 1). Experiments with a zebrafish model of mycobacterial infection demonstrated the *in vivo* efficacy of curcumin. Treatment with curcumin for 24h was associated with a significant decrease in fluorescent pixel count in *M. marinum*-infected zebrafish larvae, indicative of a lower bacterial burden in the treated animals when compared to DMSO-treated controls ( Figure 4H; ***p*<0.01**).

## Discussion

**Figure 5.**
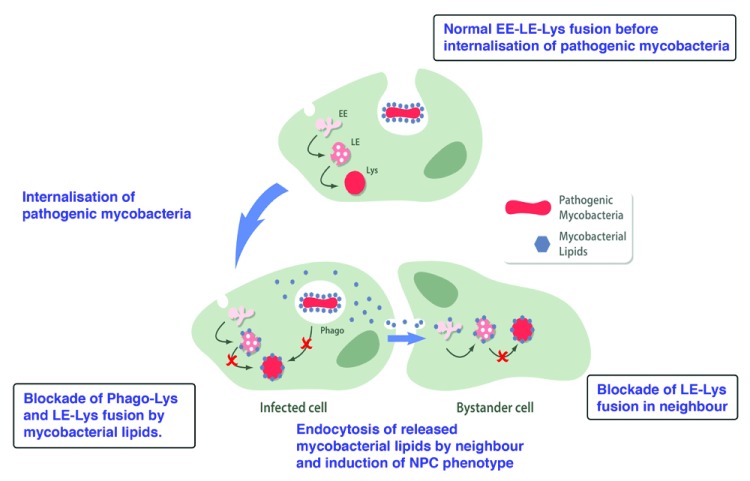
Schematic of proposed lipid-mediated inhibition of the NPC pathway by pathogenic mycobacteria. Following internalization by phagocytosis mycobacteria residing in the phagosome shed cell wall lipids, which reach the LE/Lys of the host cell where they inhibit the NPC1 protein. This causes a reduction in LE/Lys Ca ^2+^ levels and blocks phagosome-lysosome fusion. Lipids released by infected cells are endocytosed by neighboring cells and induce NPC phenotypes, including blockade of late endosome-lysosome fusion. EE, early endosome; LE, late endosome; Lys, lysosome; Phago, phagosome.

Here, we present evidence that mycobacteria are capable of preventing host phagosome-lysosomal fusion, and thereby persisting intracellularly (such as BCG and *Mtb*), may do so via lipid-mediated inhibition of the host NPC pathway ( Figure 5). The link between this rare lysosomal storage disorder and *Mtb* infection has important implications for understanding host-pathogen interactions and for developing new therapies to combat TB, particularly in this era of antibiotic resistance.

Phagocytosed *Mtb* bacilli undergo a period of rapid multiplication, concomitant with granuloma development ^4^. A significant element of the mycobacterial intracellular survival strategy is its ability to inhibit phagosome-lysosome fusion. Here, we provide evidence supporting a model in which intracellular mycobacteria, such as *Mtb* and BCG, secrete lipids that inhibit the host NPC pathway, phenocopying *NPC1 ^-/-^* cells ( Figure 5). The NPC phenotypes induced in the wild-type host cells include elevated levels of sphingosine, which in turn reduces LE/Lys-mediated Ca ^2+^ release ^22^, leading to reduced phagosome-lysosome fusion, facilitating intracellular mycobacterial survival. Pharmacological compensation for this lysosomal Ca ^2+^ homeostatic defect, by decreasing Ca ^2+^ buffering by the ER (via the action of curcumin) and subsequently elevating cytosolic Ca ^2+^ levels, enhanced clearance of pathogenic mycobacteria *in vitro* and in zebrafish infected with *M. marinum*. These findings suggest a new host-targeted approach for treating latent *Mtb* infection. Our findings also contribute to the debate on the involvement of Ca ^2+^ in phagosome-lysosome fusion and support published studies suggesting it is a Ca ^2+^ dependent process ^15^.

Induction of NPC phenotypes was not restricted to macrophages that harbour internalised mycobacteria, but was also observed in uninfected bystander cells. Cell wall-derived lipids from intracellular mycobacteria have been previously noted to be actively trafficked out of the phagosome and distributed within the infected cell, as well as within extracellular vesicles that can be endocytosed by neighbouring macrophages ^52^ ( Figure 5). We found that exposure to either the mycolic acid fraction (from BCG) or glycomycolates (mycolic acid esters) derived from *Mtb* or BCG resulted in induction of NPC cellular phenotypes in a number of wild-type cell lines, replicating the effect of the intact mycobacterium. Of the glycomycolates that were initially tested ( Figure 2G) the largest response, in terms of increased LysoTracker fluorescence (a measure of relative acidic compartment volume), was seen using TDM purified from the *Mtb* cell wall. Subsequent experiments using BCG TDM demonstrated the ability of the glycomycolate to induce NPC disease cellular phenotypes, including the lysosomal Ca ^2+^ defect, increased LE/Lys localisation of sphingosine (or reduced transport of sphingosine from LE/Lys), and accumulation of cholesterol in wild-type murine and human macrophages ( Figure 2H, I, J and K). The immunomodulatory properties of TDM (cord factor) have been previously documented, with it initiating pro-inflammatory responses ^53^ and inducing granuloma and lipid droplet formation in mice in the absence of the intact mycobacterium ^5, 33^. The importance of TDM supports previous work which demonstrated that mycobacteria possessing lower levels of the glycomycolate (either due to mutation or chemical removal) have reduced virulence and an impaired capacity to modulate endocytic trafficking and phagosome maturation ^40, 54, 55^. Probing the relationship between the structure of mycobacterial lipids/glycomycolates and their ability to induce NPC phenotypes in wild-type host cells is a complex issue. Whilst a given glycomycolate, such as TMM, can be found in the cell walls of both intracellular and environmental mycobacteria, the structure of the mycolic acid moiety of the glycomycolate will differ greatly between species ^56^. For example, mycolic acids from *Mtb* have a relatively high degree of cyclopropanation when compared to *Msm*, with 70% of mycolic acids from *Mtb* possessing two cyclopropane rings ^38^. There is also great variation with regards to the structure of the mycolic acid motifs of a given glycomycolate, even within a species. For example, the use of MALDI-TOF mass spectrometry to determine the molecular mass of the mycolic acid in *Mtb* trehalose monomycolates (TMM) revealed up to 38 significant distinct molecular species ^57^. The importance of ‘canonical’ mycolic acid structures in host cell-mycobacteria interactions is indicated by the reduction in granuloma formation induced by a mutant strain of *Mtb* unable to catalyse mycolic acid cyclopropanation ^58^.

The simplest hypothesis to explain our findings is that inhibitory mycobacterial lipids/glycomycolates directly bind to functional host cell NPC1 and inhibit its function, although an indirect mechanism cannot be ruled out. Unfortunately, a reliable binding assay for NPC1 does not exist, and there is also no direct functional assay for NPC1, making this a technically difficult hypothesis to test. However, the level of susceptibility of a cell to lipid-induced NPC phenotypes appears inversely proportional to the levels of functional NPC1 ( Figure 3A and B); *Npc1* ^+/-^ macrophages were more sensitive to TDM-induced lipid mistrafficking than their wild type counterparts, whilst NPC1 overexpression conferred resistance. Increased levels of NPC1 (but not NPC2) protein expression post-BCG infection ( Figure 3C) may reflect attempts by the host cell to compensate for reduced protein function by increasing NPC1 expression. Significantly, the NPC1 protein is also up-regulated in *Mtb* granulomas *in vivo* ^33^. This is not accompanied by an up-regulation in other lysosomal markers (e.g. LAMP1). Little is currently known about the mechanisms by which NPC1 expression is regulated. The NPC1 up-regulation we observed may slow the rate of induction of NPC disease cellular phenotypes by the mycobacterium, as we saw in the NPC1 overexpressing cells ( Figure 3B). However, the enhanced copy number of NPC1 protein will still be subject to inhibition by mycobacterial lipids, so cannot prevent the development of stable infection over time. Pharmacological or genetic blockade of NPC1 significantly enhanced the survival of non-pathogenic mycobacterial species ( Figure 3D and E). This may have significant implications for NPC patients as it suggests they are likely to have altered microbial handling, and as a result harbour an unusual microbiome, and potentially have greater susceptibly to *Mtb* infection. Indeed, altered microbial handling was recently demonstrated *in vitro* and linked to a high penetrance of Crohn's disease in NPC1 patients ^59^.

NPC1 is a mammalian orthologue of an ancient family of bacterial transporters termed Resistance Nodulation Division (RND) permeases ^19^. Interestingly, a member of this family of proteins (termed MmpL) acts as a mycolic acid transporter, facilitating lipid secretion by mycobacteria (including *Mtb*) ** ^60^ *.* A drug that targets this transporter - SQ109 - is currently in clinical trials for treating TB ^61^. Members of this conserved family of RND proteins have the ability to bind glycomycolate, with binding of Mmpl3 (essential for *Mtb* viability) to TMM previously demonstrated ^60, 62^. It may therefore be the case that the mammalian NPC1 protein also binds mycolic acids/glycomycolates, but with the lipid acting as an inhibitor not a substrate. Taken together, these studies demonstrate a remarkable role for mycobacterial RND permease family members. They are essential virulence factors for pathogen survival where they serve as mycolic acid transporters, with their mammalian counterpart NPC1 targeted by the pathogen once within the host cell ( Figure 5). The complex biology of the RND permease family of proteins remains incompletely understood and merits further investigation. Additionally, it has been proposed that free mycolic acids can assume a three-dimensional conformation similar to that of cholesterol ^63, 64^. Binding of cholesterol to the N-terminal domain of the NPC1 protein has been previously demonstrated ^65^. Mycolic acids may act as mimics of cholesterol, and in doing so bind to and inhibit NPC1.

This is the second human pathogen whose mechanism of infection has been linked to host NPC1. The second luminal loop of NPC1 serves as the first known intracellular viral receptor essential for Ebola virus infection ^66– 68^. Whether NPC1, and the broader NPC pathway, is targeted by other human pathogens (beyond Ebola and *Mtb*) is currently under investigation (Platt Lab, Department of Pharmacology, Oxford University).

Should inhibition of the NPC pathway be central to the intracellular survival of pathogenic mycobacteria, pharmacological agents that correct NPC cells may promote clearance of the mycobacterium. We did not detect enhanced microbial clearance when either HPβCD (which can ameliorate disease symptoms in animal models of NPC ^47^, most likely via stimulation of lysosomal exocytosis ^69^) or miglustat (a GSL biosynthesis inhibitor ^70^ clinically approved for NPC ^71, 72^) were tested ( Figure 4C). Whilst miglustat and HPβCD are both able to reduce cholesterol storage in NPC cells ( Figure 4A), this does not translate to a reduced intracellular mycobacterial load. It has been suggested that cholesterol storage is a downstream event in the NPC pathogenic cascade, occurring as a consequence of aberrant lysosomal fusion ^22^. Correction of cholesterol storage would therefore not be expected to lead to a restoration of lysosomal fusion. An earlier event in the pathogenic cascade is the reduced release of Ca ^2+^ from the LE/Lys. Curcumin is a SERCA inhibitor that reduces Ca ^2+^ uptake into the ER (hence increasing the availability of cytosolic Ca ^2+^) and driving lysosomal fusion ^22^. The ability of curcumin to modulate intracellular Ca ^2+^ appears key to its ability to reduce host cell mycobacterial load ( Figure 4C and 4D). Treatment with curcumin was associated with a significant reduction in host cell fluorescence, indicative of a reduced intracellular load of mCherry-expressing BCG. Curcumin is a natural product that raises cytosolic Ca ^2+^ and reduces ER Ca ^2+^. The curcumin analogue FLLL31 has no effect on cytosolic or ER Ca ^2+^ levels and also has no effect on the fluorescence of mCherry-BCG infected macrophages ( Figure 4E and F). Chelation of host cell Ca ^2+^ abrogates the beneficial effect of curcumin with regards to both improving cholesterol storage and reducing BCG levels. Interestingly, miglustat showed synergy when combined with curcumin. Miglustat’s potential efficacy as a mono-therapy merits re-evaluation over a more prolonged time course, to allow more GSL turnover to take place. The lack of effect with cyclodextrin would support the proposed exocytotic mechanism of action in NPC ^69^, which would not affect the phagosome. Finally, we have demonstrated the *in vivo* efficacy of curcumin in a zebrafish larvae model of mycobacterial infection, in which curcumin gave a significant decrease in *M. marinum* load ( Figure 4H). Curcumin may prove to be of benefit in murine models of mycobacterial infection, although this may first require issues of bioavailability to be surmounted.

In summary, we have identified an unanticipated mechanistic relationship between a rare, inherited lysosomal storage disorder and the process used by intracellular mycobacteria to subvert cellular defences. These findings provide not only an explanation for the defective phagosomal maturation observed following *Mtb* infection, but also provide a unified mechanistic framework accounting for other unexplained phenotypes in *Mtb*-infected macrophages, including cholesterol ^5^ and LacCer storage ^33^, calcium homeostatic defects ^15^, GM1 mistrafficking ^73^, elevated NPC1 expression ^33^ and bystander effects on neighbouring cells ^52^. These findings also suggest that correcting or compensating for reduced NPC1 function may offer a novel therapeutic approach for treating tuberculosis that targets the host cell and should therefore not be subject to development of resistance.

## Data availability

The data referenced by this article are under copyright with the following copyright statement: Copyright: © 2017 Fineran P et al.

The data underlying this work has been uploaded to the Open Science Framework Database, and can be accessed via https://osf.io/7r33w/ (DOI: 10.17605/OSF.IO/7R33W) ^75^.

## Supplementary material

**Supplementary Figures 1–4**

**Supplementary Figure 1. Basal [Ca ^2+^] _I_ in RAW 264.7 macrophages prior to addition of GPN.** Fura-2 350/380 ratios as a measure of the basal Ca ^2+^ (i.e. before stimulation) and maximum Ca ^2+^ released upon addition of 200μM GPN. Cells were exposed to mycobacteria (MOI 12.5) for 24h prior to Ca ^2+^ imaging. There was a minor (~7%) lowering of the basal Ca ^2+^ as a result of mycobacterial infection. However, this change was not large enough to account for observed differences in maximum Ca ^2+^ release between BCG and control/ *Msm* upon GPN addition. Mean ± SEM of n=167–311 individual cells per group. **** *p*<0.001 vs uninfected control post-GPN addition (1-way ANOVA). ##/### *p*<0.05/0.01 vs uninfected control prior to GPN addition (1-way ANOVA). GPN, glycyl-L-phenylalanine-β-napthylamide; BCG, Bacillus Calmette-Guerin.

**Supplementary Figure 2. Cathepsin C activity in control and BCG-infected RAW 264.7 macrophages.** Single-cell recordings of LysoTracker fluorescence (green) from the lumen of lysosomes released upon addition of the cathepsin C substrate GPN (200μM; indicated by the arrow) to control or BCG-infected RAW 264.7 macrophages (i/ii). No difference in rate of release of LysoTracker from lysosomes upon GPN was detected between the uninfected and infected macrophages when quantified by either linear regression of the initial rate or by exponential fit of the entire post-GPN period (iii/iv respectively). GPN, glycyl-L-phenylalanine-β-napthylamide; BCG, Bacillus Calmette-Guerin

**Supplementary Figure 3. Electron microscopy of primary human macrophages.** Cells were infected (where indicated) for 48h at MOI 12.5 in the presence or absence of 2μg/ml U18666A. A *Npc1 ^-/-^* mouse liver Kupffer cell is provided for comparison. Arrows indicate storage bodies. Scale bar, 500nm.

**Supplementary Figure 4. Effect of curcuminoids on BCG growth in broth.** The effect of curcuminoids on BCG growth in 7H9 media was measured via absorbance at 600nm. Curcumins used at 30μM. Mean ± SEM. N=3. BCG, Bacillus Calmette-Guerin.
