# Supplementary material for: Pathogenic mycobacteria achieve cellular persistence by inhibiting the Niemann-Pick Type C disease cellular pathway
Source: Wellcome Open Res. 2017 Jun 21;1:18. Originally published 2016 Nov 18. [Version 2] doi: 10.12688/wellcomeopenres.10036.2 (PMC5172425; doi:10.12688/wellcomeopenres.10036.2)
Supplement: Peer review history

# Peer review: Referee response for version 2

**DOI:** 10.21956/wellcomeopenres.12858.r23675

**Published:** 2017-07-13

**Authors:** Céline Cougoule, Catherine Astarie-Dequeker

**Affiliations:** 1 French National Center for Scientific Research, Département of Tuberculosis and Infection Biology, Institute of Pharmacology and Structural Biology (IPBS), Toulouse, France; 2 Institut de Pharmacologie et de Biologie Structurale, Centre National de la Recherche Scientifique, Toulouse, France

We have read this submission. We believe that we have an appropriate level of expertise to confirm that it is of an acceptable scientific standard.

# Peer review: Referee response for version 2

**DOI:** 10.21956/wellcomeopenres.12858.r23677

**Published:** 2017-07-03

**Authors:** Andrew P. Lieberman

**Affiliations:** 1 Department of Pathology, University of Michigan Medical School, Ann Arbor, MI, USA

The authors have adequately addressed my comments in the revised manuscript.

I have read this submission. I believe that I have an appropriate level of expertise to confirm that it is of an acceptable scientific standard.

# Peer review: Referee response for version 1

**DOI:** 10.21956/wellcomeopenres.10812.r18599

**Published:** 2016-12-19

**Authors:** Céline Cougoule, Catherine Astarie-Dequeker

**Affiliations:** 1 French National Center for Scientific Research, Département of Tuberculosis and Infection Biology, Institute of Pharmacology and Structural Biology (IPBS), Toulouse, France; 2 Institut de Pharmacologie et de Biologie Structurale, Centre National de la Recherche Scientifique, Toulouse, France

Fineman et al. authors provide a detailed description of the macrophage modification in term of lipid contents and Niemann-Pick Type C phenotype induced by mycobacterial infection. Using well-adapted methods, they fully described acquisition of the foamy macrophage phenotype and impact of NPC treatment of mycobacteria growth. Taken together, these data provide a well performed description of macrophage lipid alteration during mycobacteria infection and the potential target of this pathway as host targeted therapy that would be of interest for the scientific community working on NPC or mycobacteria.

 The text is well written and clear, the methods are exhaustive, presentation of the results clear while the discussion is quite long. I would suggest the author to shorten the discussion in order to focus on the main message of the paper.

 Major comments that must be addressed before indexation of the manuscript:

1. Figure 1 describe the acquisition of the NPC phenotype by macrophages infected by mycobacteria. Infection with different Multiplicity of Infection and different time points should be included and the results compared with positive control of NPC cells as this phenotype is not well known by the scientific community working on mycobacteria and that will read with interest this article. The authors should clarify at least in the text why the experiments are performed at different time point between the data presented in Figure 1.
2. Quantifications should be included in Figure 1G and L, Figure 2B to F and I-J, Figure 3B.
3. Only single cells are shown to illustrate the foamy phenotype, authors should show lower scale pictures illustrating larger fields of the experiments instead of a single cell.
4. My main concern is the way mycobacteria clearance is measured. Fluorescent microscopy do not allow to conclude on bacteria clearance. Colony forming unit assay should be used after infection to concluded on bacteria clearance or the text should be changed accordingly as the difference in number of bacteria in cells can reflect differences in bacteria binding and phagocytosis by macrophages or a slower growth rate.
5. Similarly in Figure 4, bacterial content is measured by flow cytometry using 2 different MOI. The authors should justify why they used 2 different MOI (10 and 100) in Figure 4B and MPOI 12.5 in Figure 2C. They should also show the SSC/FSC dot plots and the gating strategy they used to obtain the histogram shown in B. As they used high MOI such as 100, they should also provide evidence on how they evaluate macrophage cell death in their experiments. Using the histogram, data should be calculated as % of infected cells instead of MFI of the global fluorescence.
6. Data obtained by flow cytometry illustrate the fluorescence content of infected macrophages. The authors claim that drugs induce mycobacteria clearance. Difference in fluorescence may reflect a bacteriostatic effect of the drugs on mycobacteria growth. CFU experiments should be done to conclude on bacteria clearance or the text should be modified accordingly to avoid over-interpretation of the experiments.

Minor points:

1. Scale bars must be included in the pictures when missing (Figure 1B, Figure 2B-D-J, Figure 3E, Figure 4G).
2. Authors should show level of grey pictures and merge as red and blue colours are not visible on the black background.
3. Figure 4G, time point should be included, Figure 4H, illustration of mycobacteria content in zebrafish should be included.
4. Figure 4E should be shown in 4B with the corresponding absolute values instead of the normalization of the control to 1.
5. Figure 3E, in the legend, “peritoneal macrophages” is indicated while “bone-marrow derived macrophages” is written in the text. Please clarify.

We have read this submission. We believe that we have an appropriate level of expertise to confirm that it is of an acceptable scientific standard, however we have significant reservations, as outlined above.

# Response

**Published:** 2017-06-12

**Authors:** Fran Platt

**Affiliations:** University of Oxford, UK

*“The text is well written and clear, the methods are exhaustive, presentation of the results clear while the discussion is quite long. I would suggest the author to shorten the discussion in order to focus on the main message of the paper.”*

 We thank the reviewer for her comments, and accept that the discussion is quite long. However, the discussion is attempting to bridge two previously unconnected fields of research so may require more detail than is usual. We have attempted to shorten the discussion, but found it difficult to do so without detracting from the main message of the paper.

*“Figure 1 describe the acquisition of the NPC phenotype by macrophages infected by mycobacteria. Infection with different Multiplicity of Infection and different time points should be included and the results compared with positive control of NPC cells as this phenotype is not well known by the scientific community working on mycobacteria and that will read with interest this article. The authors should clarify at least in the text why the experiments are performed at different time point between the data presented in Figure 1.”*

 We thank the reviewer for her comments, and have added macrophages with pharmacologically induced NPC phenotypes cells as positive controls in Fig. 1Fi, as well as adding in text to the Figure 1 results section referencing previous experiments performed looking at lipid accumulation and intracellular calcium levels in *Npc1 *mutant cells and in those in which NPC phenotypes have been pharmacologically induced.

*“Quantifications should be included in Figure 1G and L, Figure 2B to F and I-J, Figure 3B.”*

 We kindly direct the reviewer to our response to Reviewer 2’s comment regarding quantification.

*“Only single cells are shown to illustrate the foamy phenotype, authors should show lower scale pictures illustrating larger fields of the experiments instead of a single cell”*

 We thank the reviewer for her suggestion, and have added lower magnification images that show larger fields of cells demonstrating the foamy phenotype occurring post-BCG infection (Fig. 2 panel F).

*“My main concern is the way mycobacteria clearance is measured. Fluorescent microscopy do not allow to conclude on bacteria clearance. Colony forming unit assay should be used after infection to concluded on bacteria clearance or the text should be changed accordingly as the difference in number of bacteria in cells can reflect differences in bacteria binding and phagocytosis by macrophages or a slower growth rate.”*

 We thank the reviewer for her comments. We certainly agree that the colony forming unit assay would be a worthwhile experiment, and will carry it out in the near future. We accept that host cell fluorescence is only indicative of infection level, and have stated this in the text. However, it should be noted that the experiments looking at NPC therapies (Fig 4) were performed in such a way that therapies were only applied to infected cells **after **the initial infection stage. Host cells were incubated with mycobacteria in the absence of drug for 48 hours in order to establish infection. The bacteria-containing media was then removed and the coverslips washed thoroughly, before adding media either with or without NPC therapies. This fresh media did not contain bacteria. The only bacteria remaining are those that were bound and internalized by macrophages **before **the addition of drug. It is true that differences in host cell fluorescence post-treatment may be due to the drug affecting the growth rate of intracellular mycobacteria, and not due to the effect of the drug on bacterial clearance. However, this would not be resolved by the colony forming assay.

*“Similarly in Figure 4, bacterial content is measured by flow cytometry using 2 different MOI. The authors should justify why they used 2 different MOI (10 and 100) in Figure 4B and MPOI 12.5 in Figure 2C* *.* *They should also show the SSC/FSC dot plots and the gating strategy they used to obtain the histogram shown in B. As they used high MOI such as 100, they should also provide evidence on how they evaluate macrophage cell death in their experiments. Using the histogram, data should be calculated as % of infected cells instead of MFI of the global fluorescence.”*

 The use of MOI 10 and MOI 100 in Fig 4B is used to highlight that increased levels of infection with fluorescent mycobacteria results in host cells with increased fluorescence, which can be detected via flow cytometry. The effect of NPC therapies on host cell fluorescence, indicative of intracellular mycobacterial load, was assessed using the same MOI (12.5) that we had used earlier in the paper. We have added text in the manuscript (in the section ‘Results – NPC Therapeutics Promote Clearance of Pathogenic Mycobacteria’) to emphasize this.

 Regarding calculating data as percentage of infected cell instead of MFI of global fluorescence: the difficulty with this method of data analysis is that in an infected population of cells, not each host cell is infected equally. Microscopy-based methods demonstrated that within the population some cells avoided infection completely, some were lightly infected (<5 bacteria per host cell), with some having a much greater mycobacterial burden. If the MOI was increased then the average number of bacteria per cell also increased, but there was still variability in infection level across the cell population. Flow cytometry based methods did not give a clearly delineated ‘uninfected’ and ‘infected’ peak. Rather, as the histogram in Fig 4Bi demonstrates, there is a peak representing uninfected or lightly infected cells and then a broad shoulder to that peak representing cells of increasing infectious load. It is possible to attempt to divide the histogram into ‘uninfected’ and ‘infected’ cells. During our preliminary experiments we did investigate using this form of data analysis versus analysis of MFI of global fluorescence. We found that whilst both methods of analysis resulted in the same conclusions, attempting to divide the cell population into ‘uninfected’ and ‘infected’ required a decision to be made on the part of the researcher as to where this division should occur, therefore leading to potential subjectivity. On these grounds we proceeded to analyze our data in terms of MFI of global fluorescence.

*“Data obtained by flow cytometry illustrate the fluorescence content of infected macrophages. The authors claim that drugs induce mycobacteria clearance. Difference in fluorescence may reflect a bacteriostatic effect of the drugs on mycobacteria growth. CFU experiments should be done to conclude on bacteria clearance or the text should be modified accordingly to avoid over-interpretation of the experiments.”*

 We thank the reviewer for her comment, and have modified the manuscript text (mainly in the section ‘Results – NPC Therapeutics Promote Clearance of Pathogenic Mycobacteria’) accordingly.

# Peer review: Referee response for version 1

**DOI:** 10.21956/wellcomeopenres.10812.r17934

**Published:** 2016-12-16

**Authors:** Maximiliano Gabriel Gutierrez

**Affiliations:** 1 Host-pathogen Interactions in Tuberculosis Laboratory, Francis Crick Institute, London, UK

This work investigated the link between Niemann-Pick Disease Type C (NPC) and mycobacteria. The results have important implications that link rare lysosomal storage disorders and tuberculosis.

 Experiments are very detailed, well executed and conclusions justified. Unfortunately, the authors only speculate that mycobacterial lipids can potentially inhibit NPC1 because it seems technically difficult to test. This hypothesis is definitely of interest.

 Minor suggestions for improvement in general are:

- Larger images are needed in general, they are too small and it is very difficult to observe what the authors are describing. Scale bars are missing in many panels.
- Filipin staining and blue colour is very difficult to see. I would change it to other colour or even to a LUT.
- Some of the experiments where representative images are shown would benefit of quantitative analysis in biological replicates (e.g. Fig 2B).
- Throughout the manuscript, the authors should be careful with the description of “persistent” intracellular mycobacteria, only intracellular mycobacteria would be appropriate. It is not clear if in these experiments, the mycobacteria tested are able to persist.

I have read this submission. I believe that I have an appropriate level of expertise to confirm that it is of an acceptable scientific standard.

# Response

**Published:** 2017-06-12

**Authors:** Fran Platt

**Affiliations:** University of Oxford, UK

*“Larger images are needed in general, they are too small and it is very difficult to observe what the authors are describing. Scale bars are missing in many panels.”/ “Filipin staining and blue colour is very difficult to see. I would change it to other colour or even to a LUT”*

 We thank the reviewer for their feedback. We have increased the size of the images to improve visibility, and added scale bars where appropriate. The brightness of filipin images has been increased uniformly so that the staining is easier to see.

*“Some of the experiments where representative images are shown would benefit of quantitative analysis in biological replicates (e.g. Fig 2B).”*

 Experiments performed as part of Figure 1 show that an increase in i.e. intensity of filipin staining (Fig 1Fi) translates to a quantifiable increase in levels of cholesterol stored (Fig 1Fii). A similar relationship is seen between microscopy looking at localization of GM1 gangliosides (Fig 1G) and quantification of glycosphingolipids via HPLC (Fig 1D).

*“Throughout the manuscript, the authors should be careful with the description of “persistent” intracellular mycobacteria, only intracellular mycobacteria would be appropriate. It is not clear if in these experiments, the mycobacteria tested are able to persist.”*

 We have amended the text accordingly.

# Peer review: Referee response for version 1

**DOI:** 10.21956/wellcomeopenres.10812.r17846

**Published:** 2016-12-15

**Authors:** Andrew P. Lieberman

**Affiliations:** 1 Department of Pathology, University of Michigan Medical School, Ann Arbor, MI, USA

Fineran et al. test the interesting and innovative hypothesis that pathogenic mycobacteria inhibit NPC1 function to disrupt phagosome-lysosome fusion and facilitate intracellular persistence. In support of this idea, the authors show that infection with pathogenic mycobacteria or treatment with their cell wall lipids results in a number of cellular phenotypes that also occur as a result of NPC1 deficiency. These include disrupted intracellular trafficking of lipids including GM1, sphinogomyelin and LacCer, increased filipin staining, and decreased GPN-releasable lysosomal Ca2+. Additional data show that NPC1 gene dosage affects the occurrence of these phenotypes. While the aggregate evidence supports the occurrence of similar cellular phenotypes in NPC disease and following mycobacterial infection, the authors do not show direct functional inhibition of NPC1 protein, as suggested at several points in the manuscript including the abstract, Fig 3 title and discussion. In fact, they comment in the discussion that they are not able to show direct interaction of myobacterial lipids/glycomycolates with NPC1, raising the possibility of other cellular targets. Indeed, it is well known that defects in unrelated genes (for example, SMPD1) can cause many of these same cellular phenotypes. Therefore, greater caution in interpreting the target that triggers the demonstrated effects is warranted.

 The observation that curcumin promotes mycobacterial clearance is of interest. Curcumin is a natural product with multiple suggested intracellular targets. If it is acting on ER Ca2+ stores by inhibiting SERCA channels to trigger this effect, then complementary experiments using more specific small molecules and/or genetic approaches would strengthen this conclusion.

 It would be helpful to include Npc1 deficient macrophages as a comparison in a few experiments in Fig 1 so as to inform the reader the extent to which changes following mycobacterial infection are quantitatively similar.

 Much of the data in Fig 2 are representative images. Quantification from replicate experiments would be helpful. Similarly, quantification should be included in Fig 3B.

 Several figures are missing scale bars (1B, 2B, 2D, 4G).

 It’s difficult to appreciate the cellular structures that are stained by filipin after mycobacterial infection. Co-staining with a marker of late endosomes/lysosomes would be helpful.

I have read this submission. I believe that I have an appropriate level of expertise to confirm that it is of an acceptable scientific standard, however I have significant reservations, as outlined above.

# Response

**Published:** 2017-06-12

**Authors:** Fran Platt

**Affiliations:** University of Oxford, UK

*“While the aggregate evidence supports the occurrence of similar cellular phenotypes in NPC disease and following mycobacterial infection, the authors do not show direct functional inhibition of NPC1 protein, as suggested at several points in the manuscript including the abstract, Fig 3 title and discussion. In fact, they comment in the discussion that they are not able to show direct interaction of myobacterial lipids/glycomycolates with NPC1, raising the possibility of other cellular targets. Indeed, it is well known that defects in unrelated genes (for example, SMPD1) can cause many of these same cellular phenotypes. Therefore, greater caution in interpreting the target that triggers the demonstrated effects is warranted.”*

 We thank the reviewer for his comment. Indeed, due to the lack of a reliable binding assay for NPC1 interactions, and lack of a direct functional assay (with the function of the NPC1 protein remaining contentious), the evidence that it is the NPC1 protein that is targeted during infection/lipid treatment is necessarily indirect. However, the fact that mycobacterial infection/lipid treatment induces the range of phenotypes present in NPC cells (the combination of which is unique to NPC cells) and that susceptibility to phenotype induction is inversely proportional to levels of NPC1 protein present supports a model in which it is the NPC1 protein that is the target during infection/lipid treatment.

*“The observation that curcumin promotes mycobacterial clearance is of interest. Curcumin is a natural product with multiple suggested intracellular targets. If it is acting on ER Ca2+ stores by inhibiting SERCA channels to trigger this effect, then complementary experiments using more specific small molecules and/or genetic approaches would strengthen this conclusion.”*

 We thank the reviewer for his comments. Indeed, curcumin has been implicated to be acting on multiple intracellular targets. Here we demonstrate that high-purity curcumin modulates host cell calcium, leading to an increase in cytosolic calcium and a decrease in endoplasmic reticular calcium. A related compound (tetramethylcurcumin) has no effect on calcium. The ability of a curcuminoid to modulate host cell calcium correlated with its ability to reduce intracellular mycobacterial load. Crucially, the ability of curcumin to reduce intracellular mycobacterial load was abrogated by presence of the Ca ^2+^-chelator BAPTA. This supports a model in which it is the modulation of calcium which is key to curcumin’s action.

*“It would be helpful to include Npc1 deficient macrophages as a comparison in a few experiments in Fig 1 so as to inform the reader the extent to which changes following mycobacterial infection are quantitatively similar.”*

 We thank the reviewer for his suggestion, and have added macrophages with pharmacologically induced NPC phenotypes cells as positive controls in Fig. 1Fi, as well as adding in text to the Figure 1 results section referencing previous experiments performed looking at lipid accumulation and intracellular calcium levels in *Npc1 *mutant cells and in those in which NPC phenotypes have been pharmacologically induced.

*“Much of the data in Fig 2 are representative images. Quantification from replicate experiments would be helpful. Similarly, quantification should be included in Fig 3B.”*

 We thank the reviewer for his comment, and kindly direct the reviewer to our response to Reviewer 2’s comment regarding quantification above.

*“Several figures are missing scale bars (1B, 2B, 2D, 4G).”*

 We have updated the figures so that scale bars are included. Thanks for pointing this out.

*“It’s difficult to appreciate the cellular structures that are stained by filipin after mycobacterial infection. Co-staining with a marker of late endosomes/lysosomes would be helpful.”*

 We thank the reviewer for his suggestion. The use of increased staining with filipin as a marker for endolysosomal cholesterol is a well-established hallmark of NPC cells, with filipin staining of patient fibroblasts providing a means of diagnosing NPC (Vanier, M. *Orphanet Journal of Rare Diseases *(2010) 5:16).

# Peer review: Referee response for version 1

**DOI:** 10.21956/wellcomeopenres.10812.r18486

**Published:** 2016-12-14

**Authors:** Yiannis Ioannou

**Affiliations:** 1 Department of Genetics and Genomic Sciences, Mount Sinai School of Medicine, New York, NY, USA

This is an excellent report and the authors have provided convincing evidence for their conclusions.

 The abstract is well written and appropriate.  Perhaps the title, based on the strength of the evidence provided, should be slightly more specific and instead of “…inhibiting the Niemann-Pick C disease cellular pathway” should read, “…inhibiting the Niemann-Pick C1 Protein”.

 The studies are well designed with extensive, appropriate controls and statistical analysis of the results.  The methods are “cutting edge” and the conclusions appropriate for the data presented. All experiments are properly documented in the extensive methods section providing all necessary information for others to replicate these results.

 In summary, this is an excellent move forward in our understanding of how infectious organisms hijack the cellular machinery, in this case the LE/lys/NPC compartment and manipulate it for their own survival.  Excellent!

I have read this submission. I believe that I have an appropriate level of expertise to confirm that it is of an acceptable scientific standard.

# Response

**Published:** 2017-06-12

**Authors:** Fran Platt

**Affiliations:** University of Oxford, UK

*“Perhaps the title, based on the strength of the evidence provided, should be slightly more specific and instead of “…inhibiting the Niemann-Pick C disease cellular pathway” should read, “…inhibiting the Niemann-Pick C1 Protein”.”*

 We thank the reviewer for his very supportive comments. He suggested editing the title to implicate NPC1 as the protein inhibited by the mycobacterial lipids. We agree that this is likely the case based on our data but due to the lack of a reliable binding assay for NPC1, and the lack of a direct functional assay, the evidence implicating the NPC1 protein as the point of inhibition is circumstantial and based on the relationship between the levels of NPC1 protein and the susceptibility of a cell to mycolic acid-induced lipid mislocalisation. In light of this and other reviewers comments we have left the title as it is based on these issues.
